# Ocean Acidification: Another Planetary Boundary Crossed

**DOI:** 10.1111/gcb.70238

**Published:** 2025-06-09

**Authors:** Helen S. Findlay, Richard A. Feely, Li‐Qing Jiang, Greg Pelletier, Nina Bednaršek

**Affiliations:** ^1^ Plymouth Marine Laboratory Plymouth UK; ^2^ NOAA/OAR Pacific Marine Environmental Laboratory Seattle WA USA; ^3^ Cooperative Institute for Satellite Earth System Studies, Earth System Science Interdisciplinary Center University of Maryland College Park Maryland USA; ^4^ NOAA/NESDIS National Centers for Environmental Information Silver Spring Maryland USA; ^5^ Retired From Washington State Department of Ecology Olympia WA USA; ^6^ Cooperative Institute for Marine Ecosystem and Resources Studies Oregon State University Newport Oregon USA; ^7^ Jožef Stefan Institut Ljubljana Slovenia

**Keywords:** biodiversity, carbonate chemistry, climate change, conservation, marine, ocean acidification, planetary boundary

## Abstract

Ocean acidification has been identified in the Planetary Boundary Framework as a planetary process approaching a boundary that could lead to unacceptable environmental change. Using revised estimates of pre‐industrial aragonite saturation state, state‐of‐the‐art data‐model products, including uncertainties and assessing impact on ecological indicators, we improve upon the ocean acidification planetary boundary assessment and demonstrate that by 2020, the average global ocean conditions had already crossed into the uncertainty range of the ocean acidification boundary. This analysis was further extended to the subsurface ocean, revealing that up to 60% of the global subsurface ocean (down to 200 m) had crossed that boundary, compared to over 40% of the global surface ocean. These changes result in significant declines in suitable habitats for important calcifying species, including 43% reduction in habitat for tropical and subtropical coral reefs, up to 61% for polar pteropods, and 13% for coastal bivalves. By including these additional considerations, we suggest a revised boundary of 10% reduction from pre‐industrial conditions more adequately prevents risk to marine ecosystems and their services; a benchmark which was surpassed by year 2000 across the entire surface ocean.

## Graphical abstract

In this study, employing a detailed analysis of ocean carbonate system observations, models and biological assessments, we demonstrate that by year 2020, the average global ocean conditions had already crossed into the range of the uncertainty of the ocean acidification boundary. By extending the analysis to ocean regions, we show that 4 our of 7 regions have crossed the average boundary, while all 7 have passed into the boundary uncertainties.

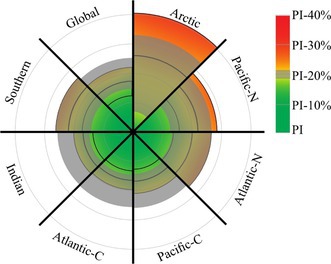

## 1 Introduction

First proposed in 2009 (Rockström et al. 2009), the planetary boundaries assessment defines nine large scale Earth‐system processes and associated boundaries that, if crossed, could generate unacceptable environmental change. These nine processes are: climate change, rate of biodiversity loss (terrestrial and marine), interference with the nitrogen and phosphorus cycles, stratospheric ozone depletion, ocean acidification, global freshwater use, change in land use, chemical pollution and atmospheric aerosol loading. Three boundaries had been crossed in 2009 (Rockström et al. 2009), increasing to four in 2015 (Steffen et al. 2015) and six in 2023 (Richardson et al. 2023). Ocean acidification (OA) was assessed as not yet having crossed the boundary, but lies at the margin of the safe operating space (Richardson et al. 2023). This remained the same conclusion in the Planetary Health Check published in 2024 (https://www.planetaryhealthcheck.org/).

OA is the term given to the long‐term shift of marine carbonate chemistry resulting primarily from the uptake of carbon dioxide (CO_2_) by the oceans (Caldiera and Wickett 2003; Orr et al. 2005), leading to an increase in ocean acidity and a decrease in carbonate ion (CO_3_^2−^) concentration. This reduction in CO_3_^2−^ influences calcium carbonate (CaCO_3_) mineral formation and dissolution (R. A. Feely et al. 2004, 2008; Gangstø et al. 2008). As CO_3_^2−^ concentration decreases, seawater CaCO_3_ saturation state (Ω) decreases, which can lead to dissolution. Conversely, when CO_3_^2−^ is plentiful, seawater is supersaturated and CaCO_3_ mineral formation is facilitated. Abiotic precipitation of CaCO_3_ minerals only occurs at very high Ω levels (Chave and Suess 1970), with the majority of CaCO_3_ in the oceans formed through biogenic processes. CaCO_3_ exists in several mineral phases, most often including aragonite and calcite, with aragonite being approximately 50% more soluble than calcite (Mucci 1983).

OA can severely affect marine organisms through its direct impact on physiology, growth, survival and reproduction (Doney et al. 2020; Findlay and Turley 2021). Furthermore, marine calcifiers that produce CaCO_3_ shells or skeletons, including some corals, crustaceans, molluscs, phytoplankton, zooplankton and algae, are at additional indirect risk from OA as decreasing Ω makes it more energetically costly to build or maintain their CaCO_3_ structures, which, when exposed to low Ω (usually undersaturated) conditions, can be subjected to enhanced dissolution (R. A. Feely et al. 2016; Findlay et al. 2011; Leung et al. 2020).

Ocean Ω conditions vary significantly across the globe, with levels in tropical regions being more than twice as high as those in polar regions (Feely et al. 2023; Jiang et al. 2015). These regional and seasonal gradients exists due to temperature‐driven CO_2_ solubility, enabling colder high‐latitude waters to store more CO_2_, along with other factors including circulation of carbon away from the surface into deeper waters, mineral inputs from land and freshwater dilution (Jiang et al. 2019; Orr et al. 2005). Marine life is exposed to such regionally varying gradients to which it has evolutionarily adapted (Vargas et al. 2022), resulting in a wide variability of observed responses to OA found in laboratory experiments. However, the envelope of the overall conditions experienced by organisms is also changing due to OA, which can make scaling up from single‐species experiments to ecosystem predictions more complicated. This is particularly true when we consider the other challenges of scaling, including incubation effects, lack of natural variability and lack of adaptation and/or acclimation.

Understanding the status, trends and biological impacts (or implications) of OA at global and regional levels is therefore paramount to determining a safe operating space at a planetary scale in which fully operational ecosystems and habitats are retained. Determining this safe space requires more than just considering chemical change. Crossing a boundary means increasing risk that marine ecosystems will be impacted by unfavourable conditions, resulting in altered ecosystem function, and ultimately cause severe implications for the societies that vitally depend on these ecosystems for a variety of provisional, cultural and climate related goods and services (Pörtner et al. 2019).

Aragonite saturation state (Ω_Arag_) has emerged as a key indicator for OA, reflecting the precipitation/dissolution tendencies of CaCO_3_, as well as its association with marine calcifiers. Consequently, the global mean surface Ω_Arag_ was chosen as the OA indicator in the planetary boundary assessments (Rockström et al. 2009). The boundary was set at 80% of the pre‐industrial Ω_Arag_ value, that is, a 20% reduction from the pre‐industrial surface ocean average. This level was chosen based on two criteria: first to keep high‐latitude surface waters above Ω_Arag_ undersaturation; and second, to ensure adequate conditions for most warm‐water coral reef systems (Rockström et al. 2009).

In the planetary boundaries framework (Richardson et al. 2023; Rockström et al. 2009; Steffen et al. 2015), the OA boundary is relatively unrefined compared to other planetary processes, which often incorporate elements of uncertainty and/or regional complexity that influences the planetary functioning. Indeed five of the nine boundaries were developed in this way during the second assessment (Steffen et al. 2015) in recognition that ‘changes in control variables at the subglobal level can influence functioning at the Earth system level, which indicates the need to define subglobal boundaries that are compatible with the global‐level boundary definition’. For example, the ‘freshwater change process’ uses the upper limit of the pre‐industrial variability as a precautionary approach, acknowledging the uncertainties related to both data and exact boundary position. While the ‘biogeochemical flows process’ has both a global and regional boundary, and the ‘land system change process’ has a global boundary as well as specific biomes boundaries (Richardson et al. 2023). In contrast, the OA boundary uses a single pre‐industrial value for Ω_Arag_ with no associated uncertainties, nor any consideration of the regional differences in manifestation of OA and the regional contribution to global ocean health and planetary functioning. This is despite Steffen et al. (2015) acknowledging that Ω_Arag_ is spatially heterogeneous, and that the criteria for defining the boundary are related to regions of the global ocean (i.e., polar waters and sub‐tropical corals), which are changing at different rates (Feely et al. 2023; Feely et al. 2024; Ma et al. 2023).

In additional to regional changes at the surface, recent research indicates that large carbonate system changes have been occurring in the subsurface (i.e., below the top 10 m routinely measured using moorings, ships‐of‐opportunities and remote sensing), where combined anthropogenic CO_2_ uptake and local respiration of organic matter interact to reduce Ω_Arag_ and pH and combine with subsurface OA‐related change (Fassbender et al. 2023; Feely et al. 2024; Harris et al. 2023; Müller and Gruber 2024). Furthermore, there is also higher frequency occurrence of subsurface compound events (marine heatwaves, decreasing DO, pH and Ω_Arag_) that synergistically impact ocean health (Gruber et al. 2021; Hauri et al. 2024).

Establishing an OA boundary that reduces the risks of significant impact and protects or sustains key marine species and ecosystems improves on a boundary that is simply defined by a chemical threshold (i.e., Ω_Arag_ = 1). The planetary boundaries framework initially addressed this for OA by considering the threshold of Ω_Arag_ for marginal growth of warm‐water coral reefs (Rockström et al. 2009). However, over the past few years, research into thresholds and indicators has developed and expanded, whereby biological impairment against changing carbonate chemistry (OA) for multiple key functional groups has been assessed through the threshold implementation (e.g., Bednaršek et al. 2019). Including additional biological indicators in the boundary assessment is especially valid given some species are found to be impacted under OA conditions in the ocean today (e.g., pteropods (Bednaršek et al. 2021; Bednaršek et al. 2012b), decapod crab larvae (Bednaršek et al. 2020), gastropods (León et al. 2020) and corals (Manzello 2010)).

Using the latest observations, modelling results and biological assessments, we explore whether setting the boundary at 20% reduction from pre‐industrial conditions provides an adequately safe limit with respect to the consequences of OA. First, we examine the latest global surface conditions in comparison to the assessment by Richardson et al. (2023), specifically using the state‐of‐art model‐data products, and importantly including uncertainties in both the boundary and the present‐day value. We also evaluate regional changes to better assess the two criteria (polar oceans and tropical corals) originally used to define the OA boundary. Next, we use new subsurface data‐model products to consider how the subsurface ocean has changed to date to acknowledge the vertical spatial heterogeneity found in the oceans. Finally, we assess these changing conditions against additional examples of OA sensitive species that serve as biological indicators, to determine what level could ultimately be considered safe for marine ecosystems and planetary functioning, including food security and carbon sequestration.

## 2 Materials and Methods

### 2.1 Models for Global and Regional Assessment

#### 2.1.1 Surface Model Data

Model simulations for the surface ocean are described by Jiang et al. (2023). They are available from (Jiang et al. 2022) as gridded products in NetCDF at the National Oceanic and Atmospheric Administration (NOAA) National Centers for Environmental Information. Data used in this analysis were the multi‐model ensemble medians and their associated standard deviations (Tables S1 and S2; [Jiang et al. 2022]).

#### 2.1.2 Subsurface Model Data

A new model‐data fusion product covering 10 global subsurface OA indicators at the standardised depth levels of 50 m, 100 m and 200 m were produced (Jiang 2024) by following the same approach as Jiang et al. (2023). These indicators include: fugacity of carbon dioxide, pH on total scale, total hydrogen ion concentration, free hydrogen ion concentration, carbonate ion concentration, aragonite saturation state, calcite saturation state, Revelle Factor, total dissolved inorganic carbon content and total alkalinity content. This product presents the evolution of these OA indicators on global surface and subsurface ocean grids with a resolution of 1° × 1°. It is presented as decadal averages for each 10‐year period, starting from pre‐industrial conditions in 1750, through historical conditions from 1850 to 2010, and extending to four future scenarios based on Shared Socioeconomic Pathways (SSPs) from 2020 to 2100. The SSPs considered are SSP1‐2.6, SSP2‐4.5, SSP3‐7.0 and SSP5‐8.5. Results for this product were extracted from 14 Earth System Models (ESMs) from the Coupled Model Intercomparison Project Phase 6 (CMIP6) and a gridded data product created by Lauvset et al. (2016). Data used in this analysis were the multi‐model ensemble medians and their associated standard deviations (Tables S1 and S2; [Jiang 2024]).

#### 2.1.3 Choice of Pre‐Industrial Value and Consideration of Uncertainties

The original OA planetary boundary used a pre‐industrial Ω_Arag_ value of 3.44, with no associated reference, however we believe this value originates from CMIP3 models, as referenced in the Royal Society report in 2005 (Raven et al. 2005), using a pre‐industrial atmospheric CO_2_ concentration of 280 ppm, which can be traced back to Caldiera and Wickett (2003). Given that atmospheric CO_2_ is used to force the ocean carbon dynamics in most ESMs, and most ESMs start their historical simulations at model year 1850, the choice of CO_2_ concentration is important.

Pre‐industrial CO_2_ concentration is derived from ice‐core records, which date back from present day to about 1000 ad. Etheridge et al. (1996) suggest that the pre‐industrial CO_2_ mixing ratio over that period is in the range of 275–284 ppm, with an uncertainty in the mixing ratios of 1.2 ppm. They also highlight ‘…Natural CO_2_ variations of this magnitude make it inappropriate to refer to a single preindustrial CO_2_ level’ (Etheridge et al. 1996). More recently the IPCC provided values for pre‐industrial CO_2_ within a range of 278.3 ± 2.9 ppm in 1750 and 285.5 ± 2.1 ppm in 1850 (Intergovernmental Panel on Climate Change (IPCC) 2023). For this reason, we take this range of CO_2_ concentrations as the pre‐industrial conditions, together with the CO_2_ uncertainties (1.2 ppm) to add an uncertainty range to the boundary rather than using one single value.

Here we use Jiang et al. (2023)'s approximation of OA indicators from 1750 and 1850, given that they are based on ice core derived atmospheric CO_2_ data from 1752 (276.39 ppm) and 1852 (288.57 ppm) (Etheridge et al. 1996; MacFarling Meure et al. 2006), and therefore represent the observed pre‐industrial CO_2_ range. Consequently, the range for pre‐industrial Ω_Arag_ is 3.44 to 3.57. Using average pre‐industrial conditions of ocean temperature, salinity and alkalinity for those dates (Table S4), we propagate the 1.2 ppm uncertainty in CO_2_ measurements to get an additional uncertainty term for Ω_Arag_ for the pre‐industrial boundary, which is 0.18 for the average global ocean, but ranges from 0.09 to 0.21 across the ocean regions. The percentage change between present day and pre‐industrial conditions and the associated uncertainty can then be calculated (section 2.1.4.). Where one single boundary value is required, for example, to calculate the change in areal extent that has crossed a specific level, the upper pre‐industrial Ω_Arag_ value (Ω_Arag_ = 3.57) is used as a precautionary level that acknowledges these uncertainties.

#### 2.1.4 Calculating Percentage Change and Propagated Errors

The percentage change in Ω_Arag_ was calculated between pre‐industrial and present‐day (2020 decade) from the multi‐model medians ($\bar{x}$ and $\bar{y}$) and their associated standard deviations ($\sigma_{x}$ and $\sigma_{y}$) using the following equations:

(1) $$\text{Percentage change}=\left(1-\frac{\bar{y}}{\bar{x}}\right)\times 100$$

The formula for error propagation of the ratio $R=\frac{\bar{y}}{\bar{x}}$ is:

(2) $${\left(\frac{\sigma_{R}}{R}\right)}^{2}={\left(\frac{\sigma_{x}}{\bar{x}}\right)}^{2}+{\left(\frac{\sigma_{y}}{\bar{y}}\right)}^{2}$$

Rearranged to:

(3) $$\sigma_{R}=\frac{\bar{y}}{\bar{x}}\sqrt{\;{\left(\frac{\sigma_{x}}{\bar{x}}\right)}^{2}+{\left(\frac{\sigma_{y}}{\bar{y}}\right)}^{2}}$$

The error in the percentage change is then: $\sigma_{R}\times 100.$

The boundary errors were calculated using the same equations, assuming $\bar{y}=0.8\bar{x}$ and, using the propagated pre‐industrial aragonite standard deviations for the boundary, $\sigma_{y}$.

### 2.2 Biological Indicator Assessment of OA Sensitive Species

Biological thresholds are defined as the inflection points beyond which detrimental biological effects are expected to begin to occur and can indicate either acute or chronic implication for the species health once the conditions have been exceeded (IOC‐UNESCO 2022). Thresholds are indispensable tools for assessing environmental conditions that may exacerbate risks for sensitive marine species and their habitats. Thresholds are not solely about achieving statistical significance, they are also about capturing ecologically meaningful responses. Such thresholds can successfully inform management and policy decisions, serving as critical communication tools for stakeholders. The drawback of such thresholds is that they do not encompass all the complexity of local adaptation and modulation introduced by simultaneous change in multiple environmental conditions (Boyd et al. 2018).

Here we combine the use of thresholds that have been determined either by strong scientific evidence from laboratory or field impacts studies, or from studies using metanalysis and expert assessment (section 2.2.1), with an environmental envelope assessment for each species (section 2.2.3) to determine a level that once crossed represents marginal conditions for that organism.

#### 2.2.1 Selection of Existing Thresholds

For the selection of thresholds it is important to understand the certainty around them. Where possible, thresholds are characterised by confidence scores, with metrics taken from the IPCC confidence model (Mastrandrea et al. 2010), and determined based on fact agreement and evidence. The confidence score ultimately delineates the level of (un)certainty around the threshold implementation, with high confidence thresholds having high certainty of the interpretation of species sensitivity and as such, a recommendation that only thresholds with medium or high certainty are to be implemented. However, in many cases when the thresholds did not undergo expert consensus, such threshold studies have not necessarily (yet) assigned confidence scores nor have a level of uncertainty associated with them. In these cases, thresholds are considered where they have been used more widely in the scientific and policy‐management communities (e.g., Barton et al. 2015; Ward et al. 2022).

Evaluating and using threshold exceedance in this study, the thresholds are taken as guidance of potential impact or vulnerability rather than absolute limit of a biological process across the global scale, reflecting a precautionary principle and recognising that nuances at the population level may alter the sensitivity of species under certain conditions. We focus on three groups that have known sensitivity to OA, are socially and economically important, and have global importance for planetary functioning: warm‐water corals, pteropods, bivalves (oysters and mussels).

We recognise that the specific driver of impacts between carbonate chemistry (OA) and the biological condition and/or biogeochemical processes are often not known, are co‐related, or a result of an indirect response. For instance, it could be pH or CO_2_, rather than Ω_Arag_ that is the main driver of impact. Due to the complexity involved in disentangling the primary drivers of the response, as well as converting between carbonate chemistry parameters (especially when not all necessary data is available within publications to do this), we present thresholds here as a function of Ω_Arag_ (Waldbusser et al. 2015) to align the chemical indicator and past planetary boundary assessments (Richardson et al. 2023).

Warm‐water coral reefs are a key indicator as they represent an invaluable ocean ecosystem. They provide habitat for a huge amount of biodiversity, hosting an estimated excess of 3 million species; they support livelihoods through tourism and fishing, providing food for over 1 billion people and a source of about 25% of the worlds fish catch; and they provide coastal protection against storms, flooding and land erosion for more than 275 million people that live near them (Spalding and Brown 2015). The threshold for warm‐water coral reefs that was already included in the OA planetary boundary assessment (Rockström et al. 2009) is used here as well. The threshold of Ω_Arag_ = 3.5 is based on the definition of the onset of marginal conditions for warm‐water coral reefs defined by Guinotte et al. (2003), derived from an environmental envelope style analysis (Kleypas et al. 1999).

Pteropods are considered key species in the polar regions with important ecosystem (Bernard and Froneman 2009) and biogeochemical significance, including making up a large component of the carbon pump (Anglada‐Ortiz et al. 2021; Manno et al. 2010), and are recognised as important OA indicators (Bednaršek et al. 2014). Present‐day levels of Ω_Arag_ in high latitudes are already causing severe pteropod shell dissolution (Bednaršek et al. 2023). The threshold for pteropods represent mild and severe shell dissolution, which serves as an early warning (mild: Ω_Arag_ = 1.5) and an indicator of additional physiological impairments (severe: Ω_Arag_ = 1.2). These shell dissolution thresholds both have high confidence scores placed on them (Bednaršek et al. 2019), and values are supported by multiple field and experimental studies both in the polar regions and the California Current Ecosystem (Bednaršek et al. 2014, 2012b).

Bivalves are included here as key indicator organisms that are critical components of coastal ecosystems. They provide a food and protein source, with bivalve production worth 20.6 billion dollars per year worldwide; they improve water quality by filtering particles, helping to balance nutrients and phytoplankton growth; they create habitats that are important nursery grounds, but also help to stabilise shorelines; finally bivalves are also important for a number of other key industries such as use in building materials, medicinal use and pearl production (Filipa Mesquita et al. 2024). OA impacts on various bivalve species have been investigated although no one specific threshold has yet been determined. A large fraction of bivalve impact studies have been conducted on larval life stages, with the onset of impacts occurring at Ω_Arag_ levels between 1.3 and 1.9. The most applied and validated impact is on the Pacific oyster (*Magallana gigas*), which has been well studied because of the impact of OA on larval production off the west coast of North America. Larval production was shown to have a negative relationship to Ω_Arag_ (Barton et al. 2012, 2015). Using this relationship, we determined the Ω_Arag_ value at which there is zero relative production and used this as a threshold (Ω_Arag_ = 1.75) beyond which relative production is minimal or does not occur. Other bivalve species, from laboratory studies, have possible sublethal thresholds related to growth and calcification (e.g., the Olympia oyster ( *Ostrea lurida* ) has onset of impacts at Ω_Arag_ of 1.4 (Hettinger et al. 2012); the Eastern oyster ( *Crassostrea virginica* ) has onset of impacts at Ω_Arag_ of 1.83 (Gobler and Talmage 2014); and the blue mussel ( *Mytilus californianus* ) has onset of impacts at Ω_Arag_ of 1.8 (Gaylord et al. 2011)).

#### 2.2.2 Geographic Distribution and Associated Environmental Envelopes

The IPBES secretariat defines an environmental envelope of a species as the set of environments within which it is believed that the species can persist. These envelopes are used in environmental niche modelling by matching habitat usage of species against local environmental conditions to determine the relative suitability of specific geographic areas for a given species (e.g., AquaMaps, (Ready et al. 2010)). The Ocean Biodiversity Information System (OBIS) database was used to gather occurrence data for each of the chosen species: *Magallana gigas* ((OBIS 2023b) and Table S5); *Mytilus californianicus* ((OBIS 2023c) and Table S6) and *Limacina helicina* ((OBIS 2023a) and Table S7). The warm‐water coral reef occurrence data was from UN Environment Programme World Conservation Monitoring Centre (UNEP‐WCMC, WorldFish Centre, WRI, TNC 2021). Data were downloaded and then sorted. Importantly noting that these datasets do not imply absence of a given species at other locations but simply represent where species have actually been observed and can be used for quantitative purposes. A secondary screening was then conducted to sanity check the data and remove duplicate records based on latitude, longitude and date of each observation. The location values were then used to extract environmental data (temperature, salinity and carbonate chemistry parameters) from the OceanSODA‐ETHZv1 dataset (Gregor and Gruber 2020). The OceanSODA‐ETHZv1.2023 dataset is a product that provides data on a 1° x 1° spatial and monthly temporal resolution between 1982 and 2022 (Gregor and Gruber 2021). Noting that only surface values are available from this dataset. Overall global environmental envelopes were generated using the nearest location match between the occurrence dataset and the OceanSODA‐ETHZv1.2023 dataset for each species. Statistics were generated from the extracted data (Tables S14–S17) and histograms (Figure S7) were generated. Analysis was conducted in R v4.1.3.

#### 2.2.3 Cross Validation of Thresholds and Environmental Envelopes

The aim of using a combined assessment is to cross‐validate these values to derive the most comprehensive interpretation of response, and hence indicator, to OA as possible. The combination of the environmental niche modelling with the threshold approach can support how information on physiological responses, derived primarily from laboratory experiments, can relate to the occurrence distribution of a species. This can give insights into when the conditions below the physiological thresholds carry over into the population absences. Such an approach is relatively novel but has important implications to detect early warning responses beyond which we would expect population level impacts to occur (i.e., when physiological thresholds overlap with the higher absence values from niche modelling).

Using the full datasets available for both occurrence and environmental data, we propose to use the 10th percentile of the environmental envelope distribution as the corresponding validation of the laboratory‐based thresholds. We use the 10th percentile to provide a standardised assessment of what can be considered extreme exposure, building on the definitions used in atmospheric and marine heatwaves and OA extremes (which use the 90th percentile for heatwaves and 10th percentile of OA (Gruber et al. 2021; Hobday et al. 2016)). The 10th percentile occurs at Ω_Arag_ = 3.5 for warm‐water corals, Ω_Arag_ = 1.1 for pteropods and Ω_Arag_ = 1.8 and Ω_Arag_ = 1.9 for the two bivalve species investigated here (*Magallana gigas* and *Mytilus californicus*, respectively) (Tables S14–S17, Figure S7).

This 10th percentile value, combined with the assessment of the thresholds in the literature, increases the confidence in the validity of these values as representing the vital biological thresholds beyond which detrimental biological effects are expected to begin to occur (IOC‐UNESCO 2022). Hereon, we use the combined assessment (considered to be the median of all the values (threshold and environmental envelopes) derived for each group) to give indicator values as: Ω_Arag_ = 3.5 as marginal conditions for warm‐water corals, Ω_Arag_ = 1.2 as marginal conditions for pteropods (but also include Ω_Arag_ = 1.5 as the mild level), and Ω_Arag_ = 1.8 as marginal conditions for bivalves.

#### 2.2.4 Application of Marginal Conditions to Biogeochemical Observational Data

Several diagnostics were then calculated using the biological assessment of the marginal conditions related to Ω_Arag_:

- The percentage of ocean area that has marginal conditions:
  ○ In the pre‐industrial era (using upper level as precautionary value)
  ○ In the present day (2020 ad)
  ○ When applying a 20% reduction in Ω_Arag_ from pre‐industrial values
  ○ When applying a 10% reduction in Ω_Arag_ from pre‐industrial values
- The change in proportion of area that is under marginal conditions between pre‐industrial and present day (2020 AD)
- The percentage reduction required from pre‐industrial before the threshold of marginal conditions was reached on average

These diagnostics were applied to each of the indicator groups using the model results for global surface waters and then the region and depth specific data using the subsurface model outputs.

From assessment of the literature and maps of each species occurrence, the regions in which the chosen indicator groups are most abundant and/or has a relevant role in the ecosystem were chosen. For warm‐water corals, they are found in the low latitudes between 40° S to 40° N at depths of 0–25 m. For pteropods, we chose two geographic regions, the polar regions (Arctic defined by the area north of 65° N, Southern Ocean defined by the area south of 45° S) and the California Current Ecosystem (defined by the geographic box of 47.5° N to 21.5° N, 108.5° W to 132.3° W (https://www.marineregions.org/gazetteer.php?p=details&id=8549), and restricting to within 300 km from the shore), at depths of 0 to 200 m (Akiha et al. 2017; Bednaršek et al. 2012a; Hunt et al. 2008; Kobayashi 1974; Zamelczyk et al. 2021). For bivalves, we chose to use the global coastal oceans (defined as within 300 km from the shore), at depths of 0–25 m (Gabaev 2015; Knights et al. 2006; Weinstock et al. 2018).

The area‐weighted mean and standard deviations of Ω_Arag_ across model grid cells within each region of interest at each depth layer (0 m, 25 m, 50 m, 100 m and 200 m) were calculated with MATLAB. The Ω_Arag_ at the 25 m depth layer was estimated as the arithmetic mean of the 0 m and 50 m layers. The area‐weighted covariance of Ω_Arag_ between layers was calculated with MATLAB using weightedcorrs (Pozzi et al. 2012). Uncertainties in depth‐integrated Ω_Arag_ accounting for covariance between layers were propagated using the delta method as implemented in the Python uncertainties library. The percentage of ocean surface area that crossed each biological threshold was calculated as the sum of the areas of all 1° × 1° model grid cells crossing the threshold divided by the total surface area in each region using MATLAB. Observed distributions of each indicator group were overlaid using data from OBIS for all species except warm‐water corals, which we took from UNEP‐WCMC (section 2.2.2). Figures S3–S6 show maps of the threshold application of pre‐industrial conditions, year 2020 conditions, 10% and 20% reductions from pre‐industrial conditions, for each of the indicator groups described above, and detailed in Tables S8 and S9. Outputs for the pteropod threshold results at each individual depth layer (0, 50, 100 and 200 m) are also shown in Tables S10–S13.

## 3 Results

### 3.1 Global Surface Ω_Arag_ as a Planetary Boundary

**FIGURE 1:**
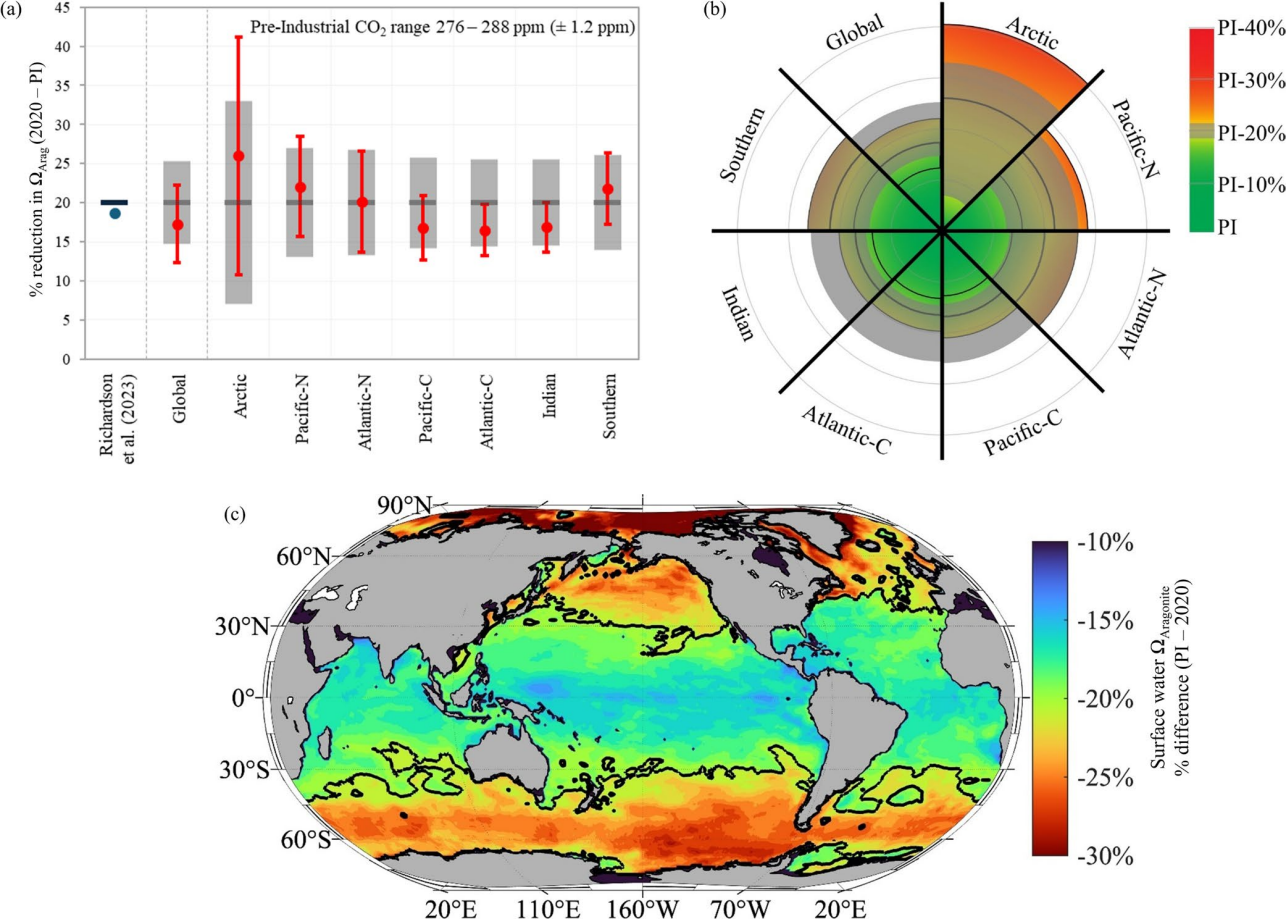
Ocean Acidification planetary boundary. (a) Percentage (%) reduction between present day and pre‐industrial aragonite saturation state for the surface global ocean and the seven ocean regions, also comparing to the Richardson et al. (2023) planetary boundary assessment (blue circle and blue line). The red circles represent the multi‐model ensemble median with the associated propagated errors for the multi‐model ensemble standard deviation and the pre‐industrial uncertainties. The 20% boundary value is presented as the dark grey lines with their associated uncertainties shown by the light grey banding. Regions are defined as: Arctic Ocean (Arctic) region north of 65° N; north Pacific Ocean (Pacific‐N) between 40° N and 65° N; north Atlantic Ocean (Atlantic‐N) between 40° N and 65° N; central Pacific Ocean (Pacific‐C) between 40° S and 40° N; central Atlantic Ocean (Atlantic‐C) between 40° S and 40° N; Indian Ocean (Indian) between 40° S and 25° N; and the Southern Ocean (Southern) ocean south of 40° S. (b) Regional assessment of ocean acidification in year 2020, relative to the boundary of 20% reduction from pre‐industrial aragonite saturation state, as in (a), with grey bars representing the boundary uncertainties, and colours depicting whether that boundary has been crossed (red) or not (green). (c) Map showing the percentage difference in surface Ω_Arag_ between pre‐industrial (1750) and year 2020. The black contour line on the map represents a 20% reduction from pre‐industrial values.

Richardson et al. (2023) estimated year 2022 global average Ω_Arag_ (= 2.8) from the climatological average value of 3.03 in year 2000 and the corresponding global decrease of 0.1 per decade given by Jiang et al. (2015). This calculation resulted in the conclusion that there had been a 19% decrease from pre‐industrial conditions (using the single pre‐industrial [1850 ad] value of 3.44) and hence, that the boundary (of 20% reduction) had not been crossed. The most recent synthesis of the global surface ocean in situ and model data suggests that present day global mean Ω_Arag_ is 2.90 ± 0.06. These updated values would suggest that OA is slightly further from crossing the boundary than Richardson et al. (2023) proposed (2.8 [19% reduction] vs. 2.90 [16% reduction]). However, incorporating uncertainty around the pre‐industrial value, and hence the boundary, gives a pre‐industrial Ω_Arag_ value of 3.51 ± 0.065 [range 3.44 to 3.57], resulting in a boundary of Ω_Arag_ value of 2.80 ± 0.05 (Table S3). These uncertainties can be propagated through to calculate the error in the percentage change over time. Including this uncertainty puts the current global average surface OA level at 17.3% ± 5.0%, which is below the boundary average, but falls well within the new boundary uncertainties (20% ± 5.3%) (Table 1, Figure 1a).

**TABLE 1:** The percentage change between pre‐industrial era (1750) and present day (2020) compared to the 20% boundary for each region, showing ± errors in the percentage change as calculated by propagating the standard deviations (see methods).

	% change between 1750 and 2020 ± propagated error				Boundary ± propagated error			
	0 m	50 m	100 m	200 m	0 m	50 m	100 m	200 m
Arctic	26 ± 15.2	25 ± 11.3	25 ± 10.0	20 ± 9.5	20 ± 13.0	20 ± 10.0	20 ± 9.4	20 ± 8.5
Pacific‐N	22 ± 6.4	24 ± 6.4	26 ± 7.8	24 ± 14.1	20 ± 6.9	20 ± 7.3	20 ± 9.0	20 ± 12.9
Atlantic‐N	20 ± 6.5	20 ± 6.8	21 ± 6.6	20 ± 6.1	20 ± 6.7	20 ± 7.1	20 ± 7.2	20 ± 7.2
Pacific‐C	17 ± 4.1	17 ± 4.5	18 ± 5.7	21 ± 11.9	20 ± 5.8	20 ± 6.0	20 ± 6.8	20 ± 9.0
Atlantic‐C	16 ± 3.3	17 ± 3.6	18 ± 5.3	19 ± 9.9	20 ± 5.6	20 ± 5.7	20 ± 6.7	20 ± 8.7
Indian	17 ± 3.2	17 ± 3.8	20 ± 5.6	21 ± 10.3	20 ± 5.5	20 ± 5.8	20 ± 7.1	20 ± 8.8
Southern	22 ± 4.6	21 ± 4.7	22 ± 5.2	20 ± 5.6	20 ± 6.0	20 ± 5.9	20 ± 6.5	20 ± 7.4
Global	17 ± 5.0	18 ± 4.5	19 ± 5.9	20 ± 10.7	20 ± 5.3	20 ± 5.2	20 ± 6.1	20 ± 7.6

In addition to model ensemble differences, regional differences in absolute Ω_Arag_ as well as the rate of Ω_Arag_ decline can contribute to variability around the average and should be accounted for. To further delineate these regional differences, we conducted a regional scale evaluation which transforms and improves global boundary estimations. Using regional data, we evaluated whether the major oceanic basins have, respectively, crossed the 20% boundary (Table 1, Figure 1a). Average surface values show that four out of seven ocean basins have crossed the boundary: The Arctic (26.0% ± 15.2% reduction), the north Pacific (22.1% ± 6.4% reduction), the Southern Ocean (21.8% ± 4.6% reduction) and the north Atlantic (20.1% ± 6.5% reduction). However, all basins have crossed the lower limit of the boundary uncertainties (Table 1, Figure 1a).

Using the upper pre‐industrial value as a precautionary value, the percentage (multi‐model median ± SD) of surface area that has crossed the 20% boundary in 2020 (compared to 1750) was over 40% ± 9.7% of the global ocean (Figure 1b), and was 86.8% ± 15.1% of the Southern Ocean, 83.6% ± 18.6% of the north Pacific, 78.2% ± 11.1% of the Arctic, 63.1% ± 22.1% of the north Atlantic, 22.9% ± 12.4% of the central Pacific, 19.7% ± 10.7% of the Indian ocean and 15.1% ± 11.5% of the central Atlantic (Figure 1b).

#### 3.1.1 Preventing Polar Oceans From Reaching Undersaturation

The first criterion used for setting the OA planetary boundary (Rockström et al. 2009) was that global average conditions would be sufficient to keep polar waters from becoming undersaturated. While nearly all of the surface polar oceans have seen an Ω_Arag_ reduction of more than 20% compared to their pre‐industrial conditions, in terms of the annual average surface Ω_Arag_ value, the chemical threshold of 1 has not yet been crossed, that is, year 2020 Ω_Arag_ (multi‐model median ± SD) is 1.49 ± 0.14 and 1.77 ± 0.04 for the Arctic and Southern Ocean, respectively. Therefore, considering only the annual average surface value, the 20% boundary does indeed prevent the polar oceans from reaching undersaturation. However, observations and models show that some regions of both polar oceans experience periods of undersaturation seasonally, and in some cases, annually in their surface waters today (Cross et al. 2018; Qi et al. 2022; Terhaar et al. 2021).

**FIGURE 2:**
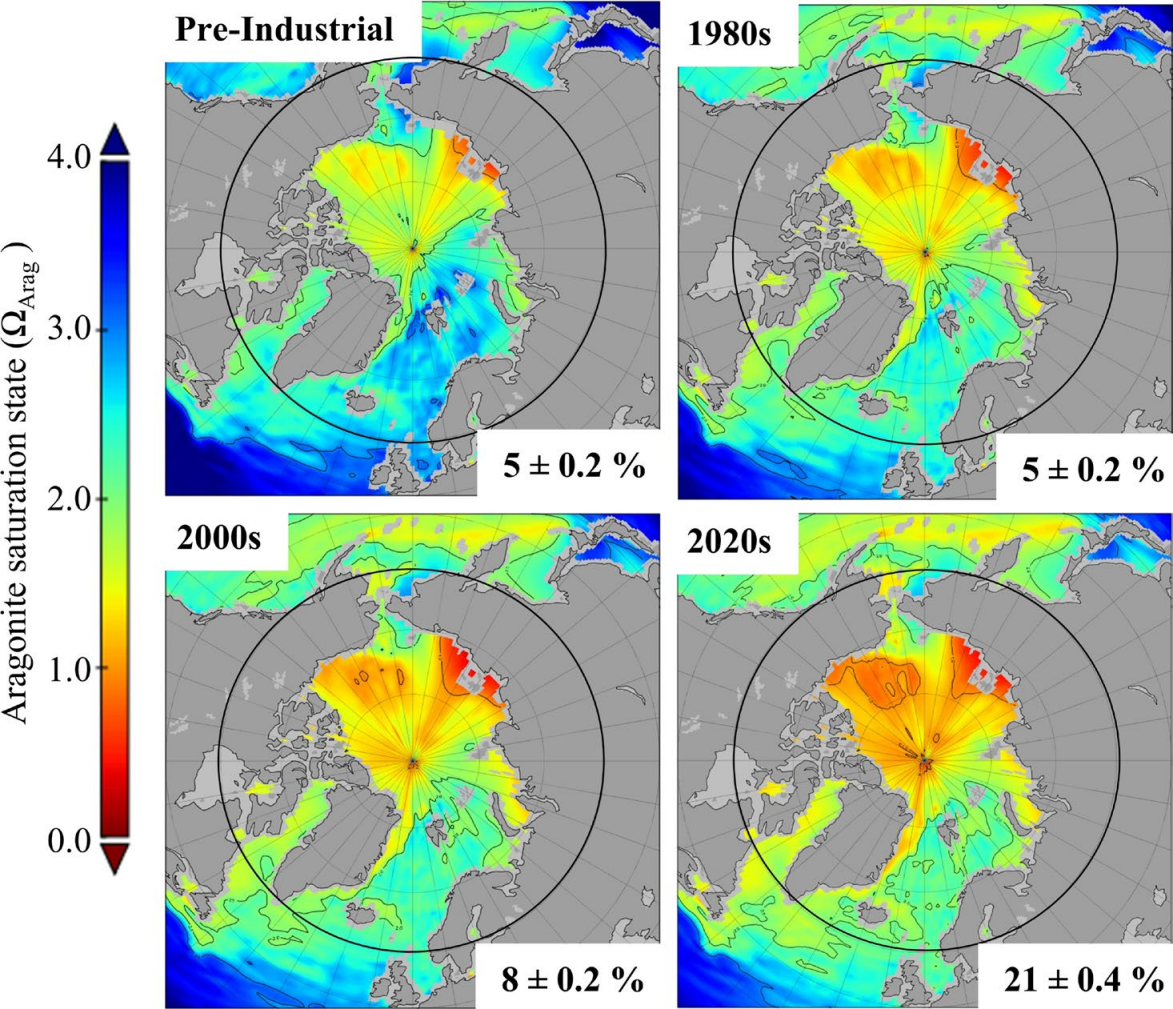
Surface water aragonite saturation state (Ω_Arag_) in the Arctic Ocean between 1750 and 2020. Maps show average conditions for the respective decade (marked at the top of each map). Numbers given at the bottom of each map shows the percentage (multi‐model median ± propagated error using multi‐model SD) of the area between 60° and 90° N that has Ω_Arag_ < 1. Maps are created using the hindcast data product from Jiang et al. (Jiang et al. 2022).

The percentage of Arctic Ocean surface waters that are undersaturated with respect to Ω_Arag_ increased between pre‐industrial conditions and 2020 ad by four‐fold (Figure 2). The model‐data product suggests about 5% ± 0.2% of the Arctic surface waters were undersaturated in pre‐industrial times, with this value remaining relatively stable until the 1980s when it started to increase. In 1990s it was ~7% ± 0.2% and in 2020s it is about 21% ± 0.2% (Figure 2). To better account for regional variability, which represents the conditions local marine organisms are exposed to, a boundary for the Arctic might be better defined by the proportion of surface ocean that is undersaturated, rather than using the absolute average surface value. For example, to keep 10% or less of the surface waters from undersaturation, the equivalent average global surface Ω_Arag_ value was passed in the late early 2000s, equating to an overall decrease of 14% ± 3.3% from the global average pre‐industrial Ω_Arag_ value. However, defining what proportion of undersaturation is within a safe margin for ecological consequences (i.e., 5%, 10%) is still subjective. Where possible, biological indicators should be included to help define safe boundaries, thus preventing biological impairment and ultimately protecting vulnerable marine ecosystems and their services.

#### 3.1.2 Preventing Tropical Coral Systems From Exposure to Marginal Conditions

**FIGURE 3:**
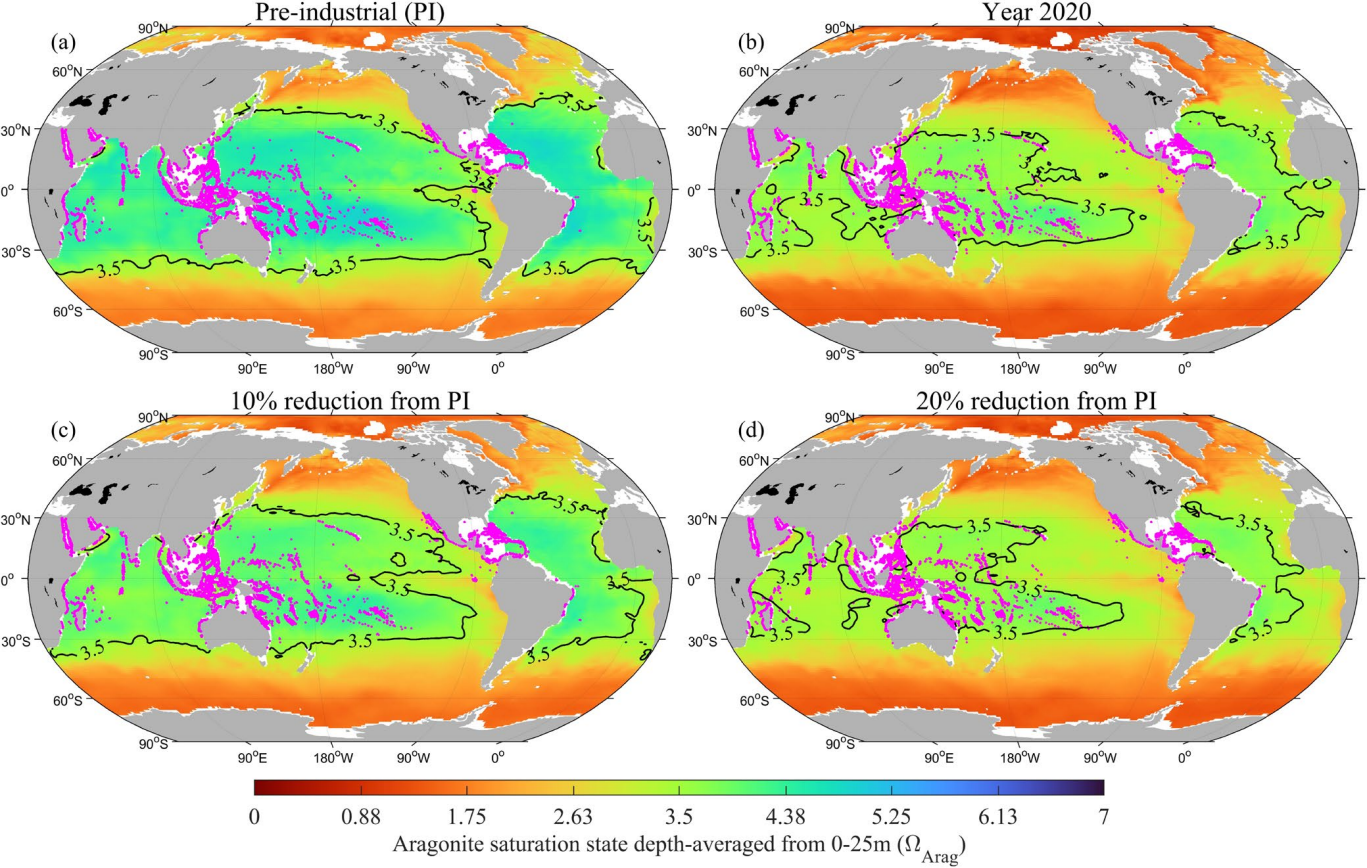
Maps of surface ocean aragonite saturation state (Ω_Arag_), highlighting the 3.5 contour to show the regions that can be considered marginal conditions for coral systems, with coral reefs distribution overlaid on each map in purple dots. (a) Pre‐industrial Ω_Arag_, (b) year 2020 Ω_Arag_, (c) Ω_Arag_ conditions at 10% reduction from pre‐industrial levels, and (d) Ω_Arag_ conditions at 20% reduction from pre‐industrial levels.

The second criterion for setting the OA planetary boundary (Rockström et al. 2009), was that the global average conditions would be sufficient to prevent warm‐water coral systems from exposure to marginal conditions (Ω_Arag_ < 3.5). Average surface Ω_Arag_ is now below 3.5 in all three low latitude (40°S to 40°N) regions which contain the highest abundance and diversity of the world's coral reefs: Year 2020 Ω_Arag_ (multi‐model median ± SD) is 3.36 ± 0.07, 3.49 ± 0.04 and 3.45 ± 0.05, for the central Pacific, central Atlantic and Indian Ocean, respectively. Hence, although the reduction in average Ω_Arag_ for each of these regions has not surpassed the 20% boundary when compared to pre‐industrial conditions, the decline in Ω_Arag_ has reached levels that represent marginal conditions for coral reef growth. To prevent these low latitude regions (taken together) from falling below 3.5, global average Ω_Arag_ should not decline more than 15% ± 9% from pre‐industrial conditions (Table 2, Tables S8 and S9). Between 1750 and 2020, the percentage area with Ω_Arag_ < 3.5 increased by 30% for the global surface ocean or 43% with respect to only the low latitude regions (Figure 3). Hence, although a large proportion of coral reefs remain in areas above 3.5 (Figure 3), the availability of suitable habitat is rapidly diminishing.

**TABLE 2:** Biological indicators. For each organism group a value for aragonite saturation state is provided that expresses marginal conditions. For the relevant range of each organism (depth and region) the aragonite saturation state value is used to calculate the percentage (%) area that has passed into marginal conditions in: Pre‐industrial (PI) conditions, 20% decline from pre‐industrial (PI‐20%), 10% decline from pre‐industrial (PI‐10%), and year 2020. Also provided is the difference (Δ%) in area between pre‐industrial and year 2020 (i.e., expansion of area that has crossed the value), and the mean ± SD (area‐weighted and depth‐integrated) aragonite saturation state reduction from the pre‐industrial conditions that can be made before crossing the value.

Species	Aragonite threshold value (ref.)	% area passed threshold, PI	% area passed threshold, PI‐10%	% area passed threshold, PI‐20%	% area passed threshold, year 2020	Δ% area passed threshold (1750–2020)	% reduction from PI before reaching threshold
Bivalve^a,c^	1.8 (Barton et al. 2012; Gaylord et al. 2011)	6.4%	10.9%	15.9%	18.6%	12.2%	51% ± 14%
Pteropod^b,d^	1.5 (Bednaršek et al. 2019)	18.7%	57.1%	75.9%	79.5%	60.8%	13% ± 16%
Pteropod^b,d^	1.2 (Bednaršek et al. 2019)	0.0%	2.8%	18.7%	15.5%	15.5%	31 ± 12%
Pteropod^b,e^	1.5 (Bednaršek et al. 2019)	0.0%	8.6%	36.5%	42.0%	42.0%	24% ± 11%
Pteropod^b,e^	1.2 (Bednaršek et al. 2019)	0.0%	0.0%	0.0%	4.0%	4.0%	39% ± 9%
Corals^a,f^	3.5 (Guinotte et al. 2003)	11.2%	27.5%	66.8%	54.0%	42.8%	15% ± 9%

*Note:* Depth ranges used: ^a^surface to 25 m; ^b^Surface to 200 m. Region used: ^c^Global coastal; ^d^Polar oceans; ^e^California Current Ecosystem; ^f^Low latitude regions (40° S to 40° N).

### 3.2 Assessing the Subsurface Ocean as Part of the OA Boundary

**FIGURE 4:**
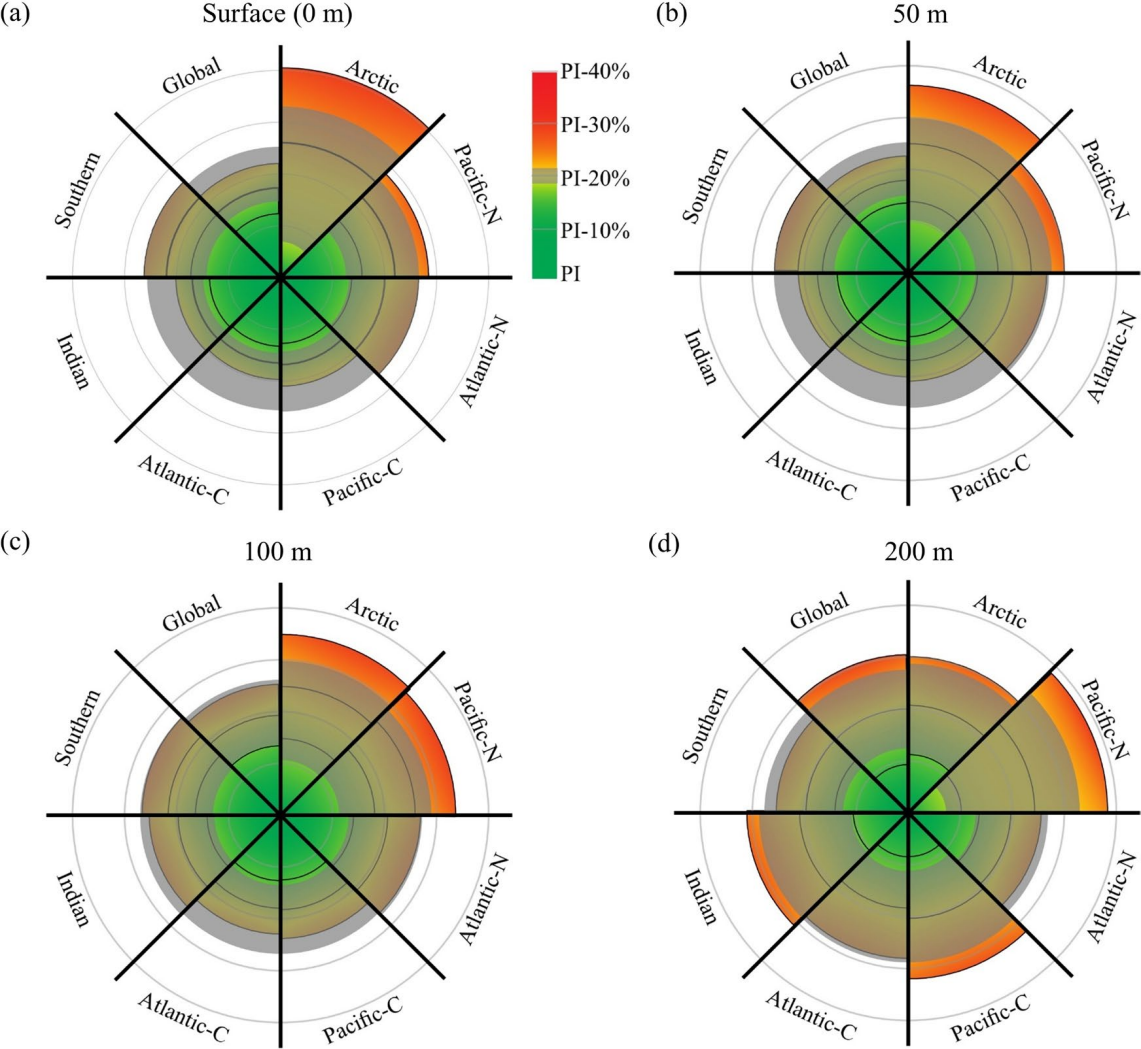
Regional assessment of ocean acidification in year 2020, relative to the boundary of 20% reduction from pre‐industrial aragonite saturation state for each depth layer. (a) surface, 0 m, (b) 50 m, (c) 100 m and (d) 200 m. The grey bands represent the boundary, the extent of each wedge represents how far each region has changed. Data is multi‐model medians from Jiang et al. (2022) and Jiang (2024) and includes ± propagated errors both on the 2020 value (black lines on each wedge) and the boundary (grey band). Regions are defined as in Figure 1.

To compare with the surface ocean, we assess the Ω_Arag_ reduction between pre‐industrial and present day (2020 ad) at three depth layers (50 m, 100 m and 200 m), including propagated errors and then calculate the proportion of area that has crossed the 20% boundary for each layer (Figure 4, Figure S1). By 2020, global average Ω_Arag_ has decreased by 17.9% ± 4.5%, 19.3% ± 5.9% and 19.7% ± 10.7% at 50 m, 100 m and 200 m, respectively. Assuming the upper pre‐industrial value as a precautionary limit, this results in 40% ± 9.7% (multi‐model median ± SD) of the surface ocean having crossed the 20% boundary by year 2020, which is about the same at 50 m (44% ± 10.8% area), but increased to 58.3% ± 10.7% area at 100 m, and 61% ± 10.6% area at 200 m (Figure 4, Figure S1).

Regionally, while the largest change at the surface has been in the polar regions, the largest change at 100 m and 200 m has been in the sub‐polar and low latitude regions (Figure 4, Figure S1). Subsurface oceans host highly diverse and biodiversity‐rich ecosystems, with few species living solely at the ocean surface. These significant subsurface changes indicate a much larger potential impact on marine ecosystems and the services they provide, including deep‐water corals, (e.g., Müller and Gruber 2024; Perez et al. 2018), pelagic fisheries and marine carbon sequestration, which need to be considered when defining the safe operating space.

### 3.3 Application of Biological Indicators

Here, we newly apply biological thresholds for the key indicator groups defined previously, that have known sensitivity to OA and are of global relevance (Table 2 & Table S8).

#### 3.3.1 Exceeding Marginal Conditions That Support Polar Food Webs

The percentage of ocean area in the polar regions, averaged across the pteropod depth habitat (0–200 m), that has crossed the thresholds for mild (Ω_Arag_ = 1.5) and severe (Ω_Arag_ = 1.2) shell dissolution, has increased by 61% and 16%, respectively, between pre‐industrial and present day (2020 ad) (Table 2, Tables S8 and S9). Furthermore, a larger percentage area of the polar oceans has exceeded the mild dissolution threshold of 1.5 by year 2020 compared to when considering the OA boundary of 20% reduction from pre‐industrial Ω_Arag_ conditions (80% vs. 76%, respectively; Table 2, Tables S8 and S9). To remain above the mild and severe dissolution thresholds, average polar ocean Ω_Arag_ (0–200 m) conditions cannot decline more than 13% ± 16% and 31% ± 12%, respectively, from the pre‐industrial value, and even at this level there will still be some areas where the threshold is crossed (Table 2, Tables S8 and S9).

#### 3.3.2 Exceeding Marginal Conditions That Support Aquaculture and Shellfisheries

The bivalve threshold of marginal conditions was taken to be Ω_Arag_ < 1.8. Using this value together with their distribution along the global coastal regions (set to < 300 km offshore and a depth range of 0–25 m), the area that has crossed into marginal conditions has increased by 12% between year 1750 and year 2020 (Table 2, Tables S8 and S9). A boundary set to 20% reduction from pre‐industrial conditions results in 16% of the global coastal habitats being marginal for bivalves. To avoid moving into marginal conditions for bivalves, average coastal Ω_Arag_ (0‐25 m) conditions cannot decline more than 51% ± 14% from pre‐industrial values.

### 3.4 Halting the Trend and Maintaining a Safe Operating Space

The unequivocal driver of OA is the rapid uptake of anthropogenic CO_2_ by the oceans. Model projections show that only by following the low emissions scenario (SSP1‐2.6) can some parts of the global surface ocean be kept within the 20% boundary, and by the end of the century those areas begin to expand again (Figure S2). This is in sharp contrast with the intermediate (SSP2‐4.5) and high emissions scenarios (SSP3‐7.0), which both lead to 100% of the global surface ocean crossing the 20% boundary. Indeed, by year 2100, nearly 25% of the global surface ocean will have Ω_Arag_ levels that are > 40% lower than pre‐industrial conditions under emissions scenario SSP2‐4.5, whereas nearly 95% of the surface ocean will have Ω_Arag_ conditions that are > 40% lower than pre‐industrial levels under SSP3‐7.0 (Figure S2).

## 4 Discussion

This study significantly advances the work of Richardson et al. (2023) by conducting a more in‐depth and refined analysis of the OA planetary boundary. It addresses limitations of previous assessments, incorporates updated scientific information, utilises additional biological indicators, and formally accounts for uncertainties. This leads to a revised and more accurate, ecologically sound definition of the OA planetary boundary. The main advancement lies in shifting from an assessment based primarily on the changing chemistry to a more holistic approach that considers uncertainties, regional variations, subsurface impacts and the biological consequences of exceeding the boundary.

Taking into consideration the uncertainties from global model ensembles, regional variability and uncertainties associated with the pre‐industrial value, gives propagated uncertainty of ±5.3% on the 20% boundary. By using both a global and regional analysis we demonstrate that large parts of the ocean have expanded into, and sometimes well beyond, these boundary uncertainties. This work is a first attempt at adding uncertainty to the OA planetary boundary. Additional errors in data collection, model development and pre‐industrial values could lead to even larger uncertainties. This is highlighted by the variability in uncertainties across the oceanic regions. For instance, the Arctic Ocean boundary has the highest uncertainties (±13%) predominantly due to the variability between models and their representation of OA dynamics in the region. There has been an improvement between the CMIP5 and CMIP6 models in terms of OA parameters in the Arctic (Terhaar et al. 2021), but it remains clear that there are still issues with the paucity of data in that region, together with important drivers of carbon cycling, such as river fluxes (e.g., Tank et al. 2023) and sea ice interaction (Qi et al. 2022; Swoboda et al. 2024), being poorly represented in these global models.

Understanding the regional dynamics is conceivably more useful than the global average in terms of the impact on functioning of the marine ecosystem, feedback to planetary systems, as well as the ecosystem services provided. For example, polar and sub‐polar regions are important for carbon uptake (Friedlingstein et al. 2024), and the interaction between OA and future CO_2_ uptake is an important planetary feedback to understand and assess (Chikamoto et al. 2023; Gehlen et al. 2011). Moreover, increasing water temperatures (included in the saturation state calculations here), which are not uniform across the global ocean, promote the dissociation of bicarbonate ions, releasing extra carbonate ions and slightly counteracts the decreasing trend of seawater saturation state (Figure S4; Jiang et al. 2019). However, this temperature‐driven change in saturation state is relatively minor, amounting to only about a 1% increase with a 5°C rise in water temperature. Furthermore, understanding key biological indicators that are fundamental parts of the food web is globally relevant, but regionally specific, given that species are shifting their biogeographic distributions with warming (IPCC 2021). Indeed, climate‐induced changes in food webs, particularly moving into the polar regions, may not be supported if OA causes a restructuring in the base of that food web. Assessing the regional conditions is also especially important if a boundary is set based on regionally‐specific criteria, and where regions are changing at different rates, as is the case here. Indeed, the regional assessment shows that although the polar oceans are not yet undersaturated with respect to average Ω_Arag_, they have crossed the 20% boundary and concur with polar observational studies that there are some regional and seasonal exceptions even today. The proportion of Arctic surface area that is undersaturated is growing rapidly and resulting in relevant biological thresholds being exceeded, demonstrating extensive species impairments under current conditions. In contrast, the low latitude regions have, on average, already transitioned into marginal conditions for supporting coral reef growth, with projected expansion of these areas, but have not yet crossed the 20% boundary.

Coastal regions are naturally more variable than the open ocean, with complex interacting drivers that are poorly constrained in global ESMs. Therefore, the uncertainties associated with changes in the coastal regions are likely to be much larger and underestimated in this work. Our analysis of the global coasts suggests that the OA signal still results in a reduction in suitable habitat for economically important calcifying species (i.e., Feely et al. 2024). The analysis here does not consider extreme events or abrupt shifts in conditions, which could be more damaging to local populations than the longer‐term chronic changes. This is known to be the case for temperature, where population die‐offs have been observed as a result of marine heatwaves (Smale et al. 2019). However, the only example, to date, of population die‐offs due to periodic OA events is the Pacific oyster larvae on the west coast of North America, where natural upwelling combined with OA has resulted in increased frequency and intensity of OA events impacting hatcheries in the region (Barton et al. 2012, 2015). Although some variability is inherently included in the work here through the model‐data uncertainty propagation, these events would add even more variability and ultimately could result in earlier exceedance of critical conditions and enhanced biological implications. Future work should include improved coordination between chemical and biological studies, as well as assessing higher resolution temporal environmental data to properly capture the environment that organisms are exposed to, including the frequency and extent of extreme conditions.

Large portions of the subsurface have already changed significantly from pre‐industrial conditions. This was recently highlighted in a paper that reconstructed ocean interior acidification over the industrial era, confirming that significant changes are occurring in the interior ocean due to the uptake of anthropogenic CO_2_ (Müller and Gruber 2024). Indeed, in addition to the horizontal spatial squeeze at specific depth ranges that is highlighted here, Müller and Gruber (2024) emphasise that shoaling of the aragonite saturation horizon (Ω_Arag_ = 1) has occurred in some places by more than 200 m, which is therefore causing a vertical squeeze on ‘safe’ habitat for many species. For example, the proportion of habitat that has passed marginal conditions at each individual depth layer for pteropods (1.2 threshold) is 16.4% by year 2020 at the surface, 13.6% at 50 m, 23.4% at 100 m and 42.7% at 200 m (full results in Tables S10–S13). Such vertically stratified information is especially useful for comparative purposes of species that occupy various depth layers to establish the extent of the vertical habitat squeeze and determine their relative sensitivity. The regional variability in shoaling (largest amount of shoaling in the Southern Ocean and North Atlantic, least amount of shoaling in the North Pacific) highlights again the complexity of OA in the 3‐dimensional space of the ocean compared to the 2‐dimensional surface. This vertical and horizontal squeeze in the chemistry needs to be recognised in assessing planetary biogeochemical functioning, feedbacks to the carbon cycle, habitat suitability and ecosystem stability.

Loss of ecosystem function or suitable habitats can lead to fragmentation, the breaking up the continuous distribution of a species into smaller, isolated patches. This fragmentation directly reduces population connectivity, as individuals within the fragmented habitats have reduced opportunities for interaction, mating and dispersal. Reduced connectivity limits gene flow between populations, which are essential for maintaining genetic diversity and sustaining adaptation potential, whereby isolated populations with restricted gene flow are more susceptible to inbreeding and reduced evolutionary potential (Bertness and Gaines 1993). Fragmentation can also limit larval dispersal, reducing the ability to seed populations, with isolated populations become increasingly vulnerable to local extinction. As such, maintaining population connectivity is crucial for ensuring the long‐term survival of marine species. More accurate OA boundary assessment as demonstrated in this study not only supports decisions on climate mitigation but can help in devising conservation strategies (e.g., Nissen et al. 2024), for example, by providing a stronger scientific foundation for setting targets within policy agreements, such as the Biodiversity Beyond National Jurisdiction (BBNJ) agreement, as well as Kunming‐Montreal Global Biodiversity Framework (CBD 2022).

Based on our new analysis of uncertainties, surface and subsurface changes and crossing into marginal conditions for key biological indicators, we propose that if a single value is to be used as an OA planetary boundary, it should be set at a more conservative value of 10% decline from pre‐industrial average global surface Ω_Arag_ conditions, rather than 20%. A boundary set to 10% pre‐industrial conditions will: (1) Limit the area of Arctic surface ocean that is undersaturated to less than 10%; (2) Sustain polar habitats and protect sensitive species such as pteropods from shell dissolution, that is, using this lower boundary, 57% of the upper 200 m of polar pteropod habitat will be at or below conditions that result in mild shell dissolution, with only 3% of the habitat space at or below conditions that result in severe dissolution; (3) Preserve conditions in tropical regions above the level required for adequate coral growth: limiting the areal loss of suitable coral habitat to 28% of the low latitude regions (Figure 3); and (4) Sustain economically and ecologically relevant bivalves in the coastal regions, not just protecting them against OA but also increasing their resilience to other stressors, including warming and deoxygenation. The percentage of global coastal habitat being unsuitable for oyster production falls to just 10% if a 10% boundary is used.

The 10% boundary is a more stringent and ecologically meaningful target, reflecting the findings of this study that 20% reduction provides insufficient protection of many crucial ocean habitats beyond the surface waters. This boundary of 10% should be considered as the lower end of an uncertainty range of increasing risk, especially important as OA should be considered in combination with other stressor and extreme events that can cause critical habitat and biodiversity loss, and restructure ecosystems. However, redefining the OA boundary to 10% means that the boundary was first crossed during the 1980s, with the entire surface ocean having passed this boundary by the 2000s. Preventing further OA increase and minimising risks to ocean ecosystems on a global level can only be done by reducing CO_2_ emissions along with rapid atmospheric greenhouse gas removal (Lee et al. 2023).

It is important to recognise the limitations of an OA boundary that only uses aragonite, as mentioned by previous critiques of the planetary boundary assessment (Biermann and Kim 2020; Brewer 2009; Nash et al. 2017). Ω_Arag_ is just one parameter of several that represent how ocean chemistry is changing in relation to OA. For instance, some biological and biogeochemical processes (e.g., primary production, carbon fixation, nitrogen cycling) have been shown to be influenced by shifts in pCO_2_ or pH (Findlay and Turley 2021). Considering how these processes, and importantly their interactions, relate to maintaining a safe operating space is a complex task, especially given many of the results come from studies that only look at response to present day and future conditions, with little information about response to pre‐industrial levels, natural variability or through recent history. As more field studies and monitoring data become available some of these gaps could be filled, and a future assessment of the OA boundary may be able to bring in these aspects as well as improve on our uncertainty assessment. In fact, it may be more pertinent to focus on the CO_2_ level that drives OA‐related change rather than pick one specific OA chemical indicator (Ω_Arag_). Indeed, as recognised by others (e.g., Rose et al. 2024), OA should not be the only marine process considered in the context of planetary boundary framework. For example, ocean warming, including marine heatwaves, and deoxygenation have wide‐scale repercussions for ocean health and planetary system functioning. The complex interactions between these drivers also needs to be considered as they manifest on different time‐frames, to varying degrees in different locations, and can result in different responses in the ecosystem when considered together, in contrast to when considered in isolation (Alter et al. 2024).

To complement the assessment of which parameter to use, further fundamental work is required to better characterise biological indicators and quantify their uncertainties, where possible taking into account life‐stage specific sensitivities, pre‐exposure conditions and adaptation strategies, recognising additional conditions that could impact the thresholds and their durations. While the thresholds used here do not cover these issues specifically, they represent the level at which potential harm may occur, in keeping with the planetary boundary framework of remaining within a safe space. Further developments of the environmental envelope assessment, to complement the threshold assessment, could include using higher resolution data that can improve the representation of exposure conditions both in space (horizontally and vertically) and in time (sub‐monthly). Indeed the environmental envelopes defined here may underestimate the range of Ω_Arag_. An underestimate will result in a more precautionary ‘limit’, but is more representative of large‐scale averages relevant for the planetary boundary framework.

We conclude that this study provides a more robust and nuanced scientific basis for the OA planetary boundary framework, although further developments, as outlined above, should be considered. This framework is being used in policy decisions related to OA, which provide the scientific basis for national and international collaboration and action, including informed prioritisation of marine conservation efforts. Regions and species most vulnerable to OA can be targeted for specific conservation measures. The subsurface impacts, in particular, require a shift in focus to protect mesopelagic and deep‐sea habitats and the species dependent on them. The incorporation of uncertainty in the study highlights the need for adaptive management strategies to deal with OA, the potential benefits of which are improved resource management and increased resilience.

## Author Contributions

**Helen S. Findlay:** conceptualization, data curation, formal analysis, funding acquisition, investigation, methodology, project administration, visualization, writing – original draft, writing – review and editing. **Richard A. Feely:** conceptualization, funding acquisition, validation, writing – review and editing. **Li‐Qing Jiang:** data curation, formal analysis, funding acquisition, methodology, visualization, writing – review and editing. **Greg Pelletier:** data curation, formal analysis, methodology, visualization, writing – review and editing. **Nina Bednaršek:** data curation, formal analysis, methodology, writing – review and editing.

## Conflicts of Interest

The authors declare no conflicts of interest.

## Supporting information


Appendix S1.

## Data Availability

The data that support the findings of this study are openly available from figshare at https://doi.org/10.6084/m9.figshare.28861832. Ocean acidification indicators were obtained from NOAA National Centers for Environmental Information at https://doi.org/10.25921/9ker‐bc48 and at https://doi.org/10.25921/vdnc‐q403. The OceanSODA‐ETHZv1.2023 data were obtained from NOAA National Centers for Environmental Information at https://doi.org/10.25921/m5wx‐ja34. Occurrence data was obtained from the Ocean Biodiversity Information Service mapper at www.obis.org. The warm‐water coral reef occurrence data were obtained from UNEP‐WCMC, WorldFish Centre, WRI, TNC at https://doi.org/10.34892/t2wk‐5t34.
