# Supplementary material for: Ocean Acidification: Another Planetary Boundary Crossed
Source: Glob Chang Biol. 2025 Jun 9;31(6):e70238. doi: 10.1111/gcb.70238 (PMC12147064; doi:10.1111/gcb.70238)
Supplement: Supplementary file 1 — Appendix S1. [file GCB-31-e70238-s001.docx]

Supplementary Materials for

**Ocean Acidification: Another Planetary Boundary Crossed**

Helen S. Findlay^1,2*^, Richard A. Feely^3^, Li-Qing Jiang^4,5^, Greg Pelletier^6^, Nina Bednaršek^7,8^

^1^Plymouth Marine Laboratory, Plymouth, PL1 3DH, UK

^2^University of Exeter, Exeter, Devon, EX4 4SB, UK

^3^NOAA/OAR Pacific Marine Environmental Laboratory, Seattle, WA 98115, USA

^4^Cooperative Institute for Satellite Earth System Studies, Earth System Science Interdisciplinary Center, University of Maryland, College Park, MD 20742, USA

^5^NOAA/NESDIS National Centers for Environmental Information, Silver Spring, MD 20910, USA

^6^Retired from Washington State Department of Ecology, Olympia, WA, United States.

^7^Cooperative Institute for Marine Ecosystem and Resources Studies, Oregon State University, Newport, OR 97365, USA

^8^Jožef Stefan Institut, 1000 Ljubljana, Slovenia

*Corresponding author. Email: hefi@pml.ac.uk

**The PDF file includes:**

Figs. S1 to S6

Tables S1 to S14


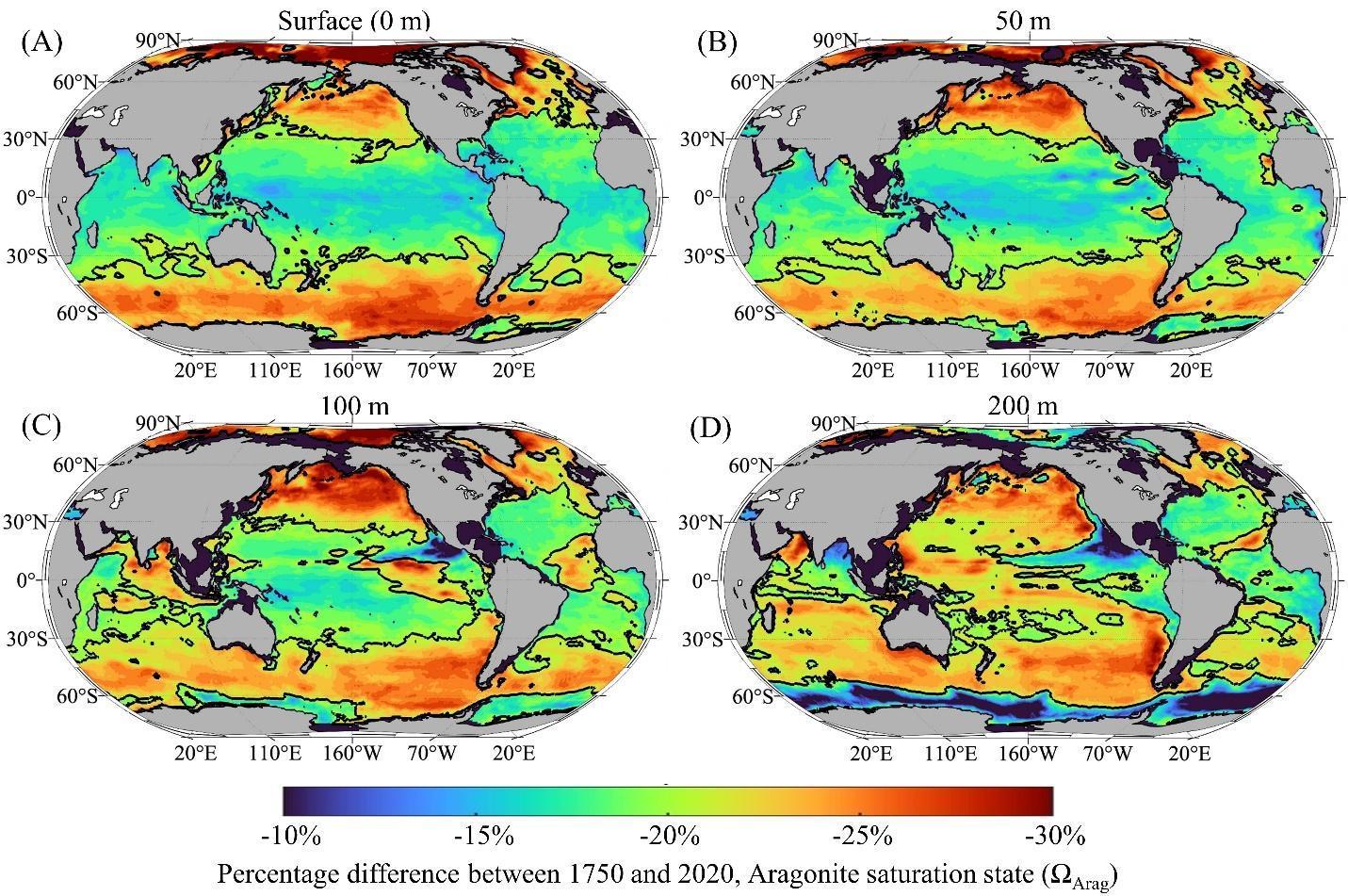
 **Fig. S1.** Maps of the percentage reduction in aragonite saturation state between the pre-industrial year 1750 and year 2020 at four different depth layers. A) surface (0 m), B) 50 m, C) 100 m, D) 200 m. The black contour line represents a 20% reduction from pre-industrial values.


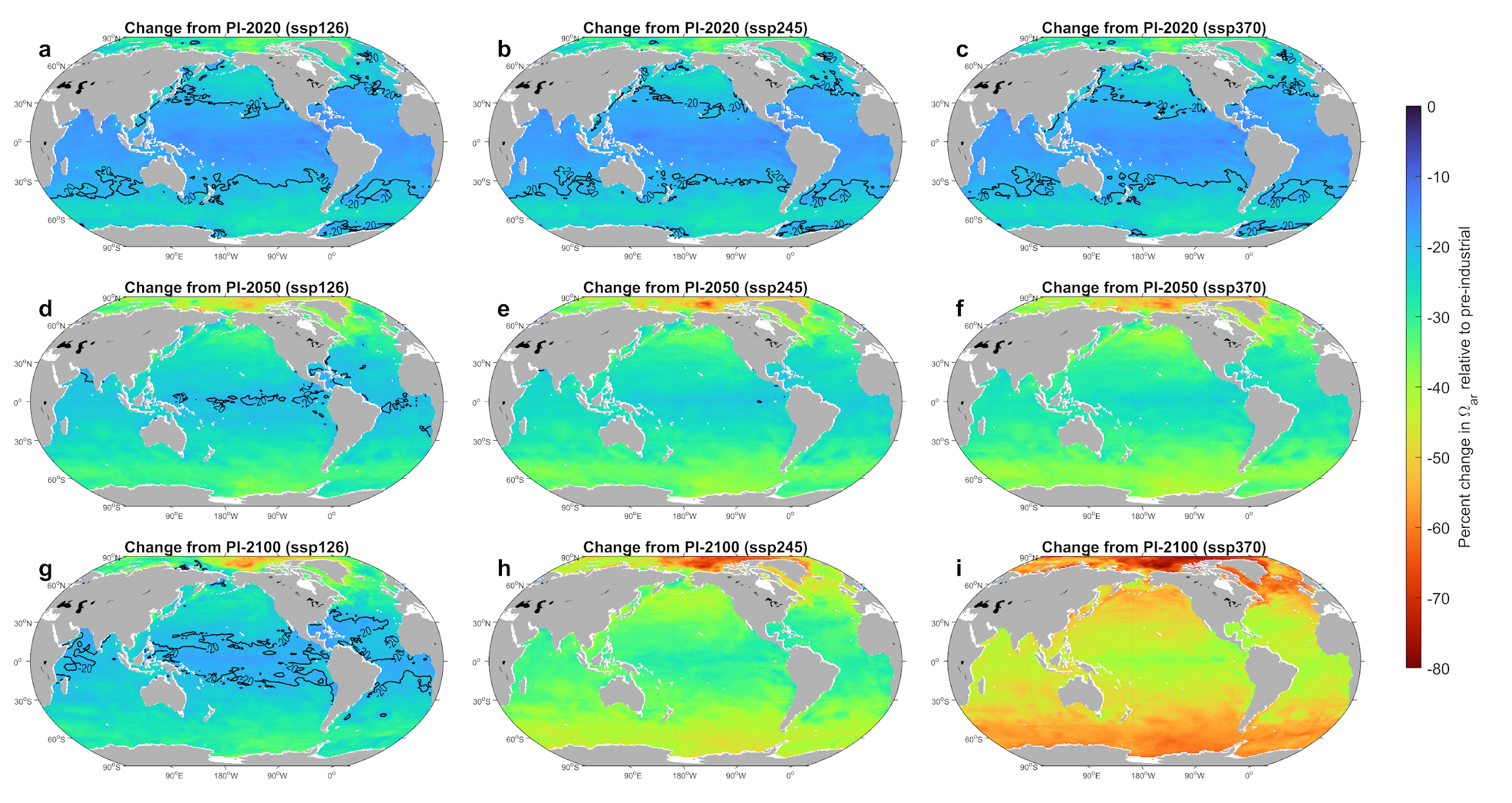


**Fig. S2**: Maps of the change in surface ocean aragonite saturation state (Ω_Arag_) between pre-industrial (PI) conditions in year 1750 and year 2020 (A-C), 2050 (D-F) and 2100 (G-I) under three CO_2_ emissions scenarios: low emissions SSP126 (A, D, G), mid-emissions SSP245 (B, E, H), and high emissions SSP370 (C, F, I).


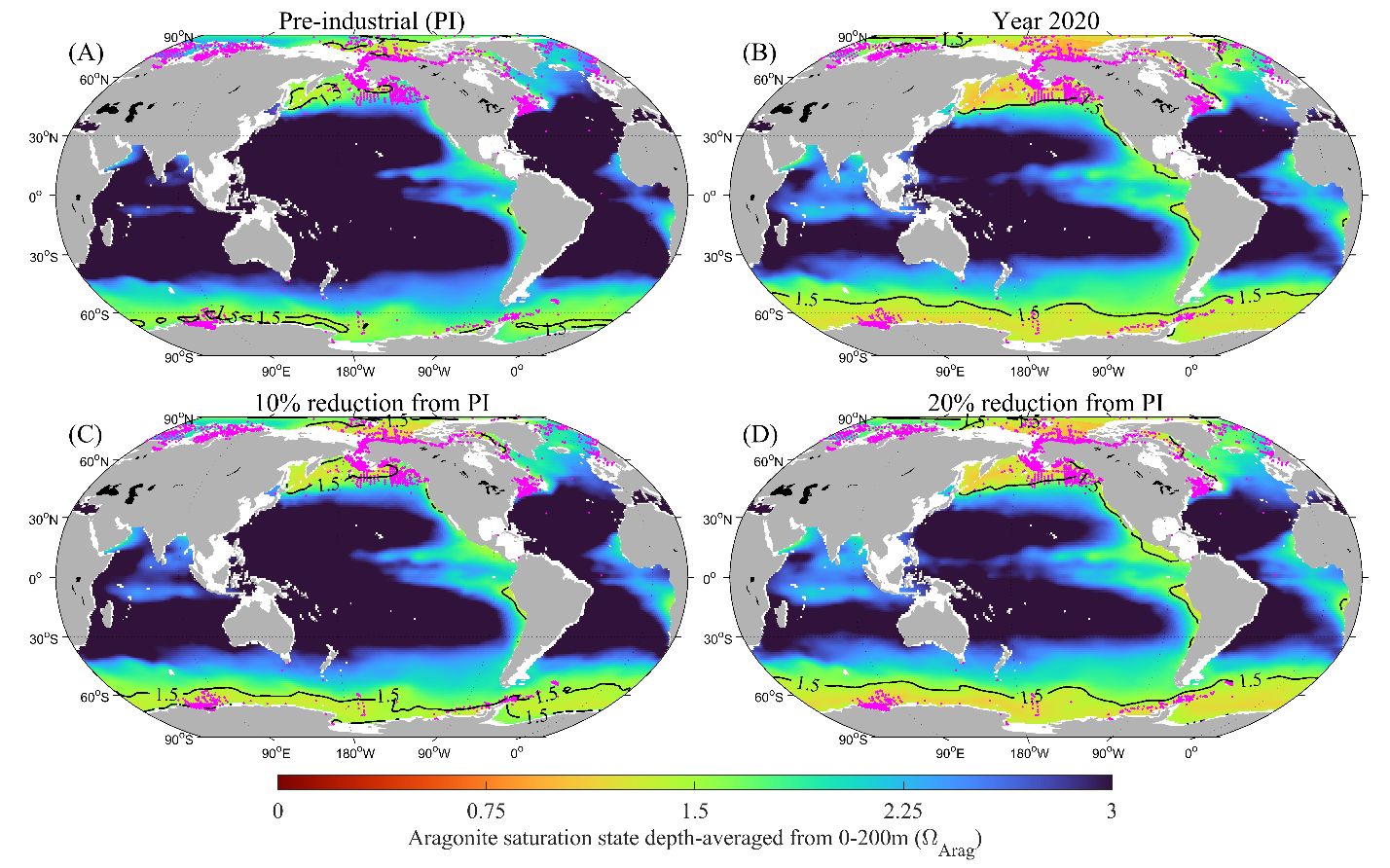


**Fig. S3**: Maps of surface ocean aragonite saturation state (Ω_Arag_), highlighting the 1.5 contour to show the regions that can be considered at risk of mild shell dissolution for pteropods, with their distribution (data from OBIS) overlaid on each map in purple dots. A) Pre-industrial aragonite saturation state, B) year 2020 aragonite saturation state, C) aragonite saturation state conditions at 10% reduction from pre-industrial levels, and D) aragonite saturation state conditions at 20% reduction from pre-industrial levels.


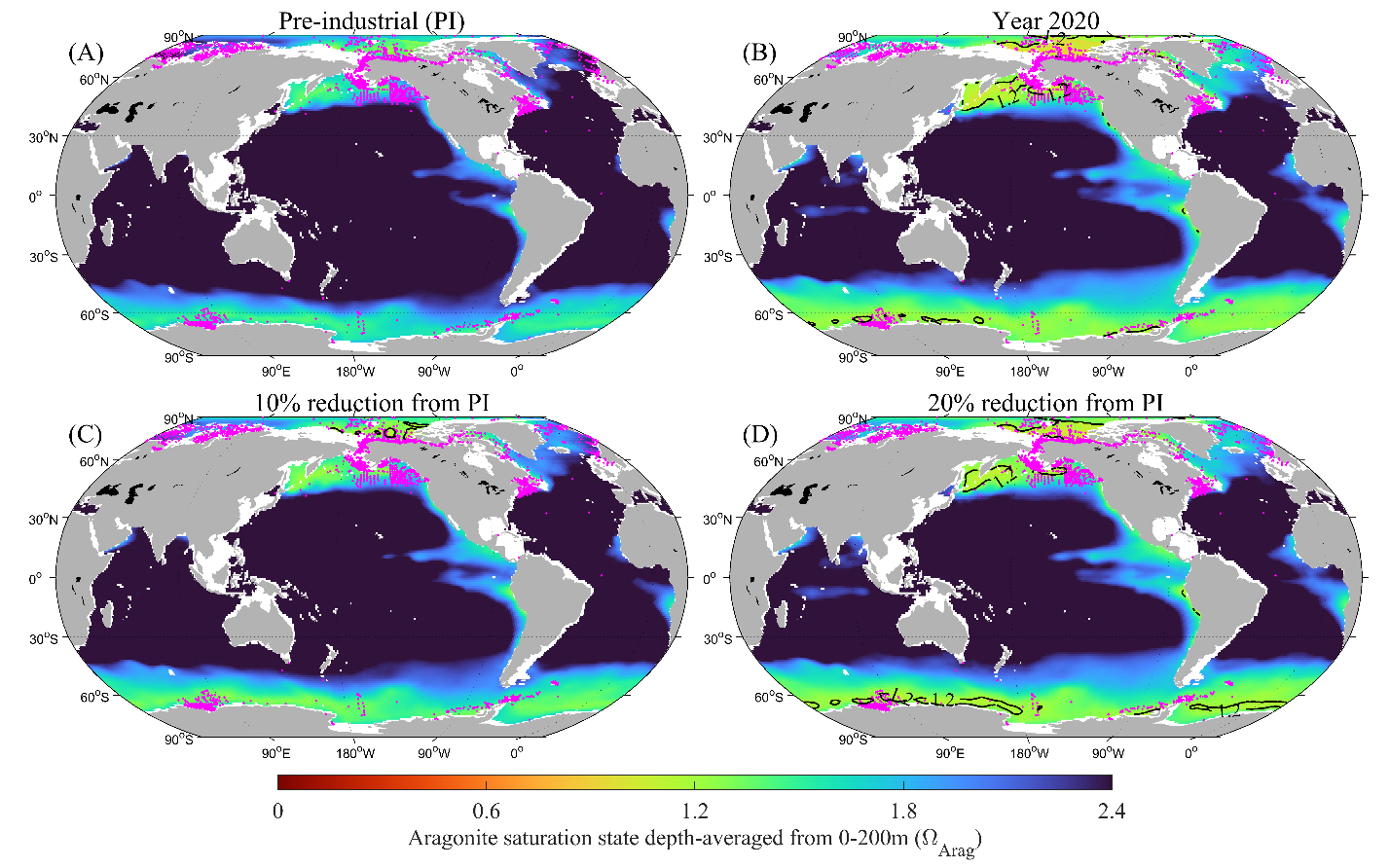


**Fig. S4:** Maps of surface ocean aragonite saturation state (Ω_Arag_), highlighting the 1.2 contour to show the regions that can be considered at risk of severe shell dissolution for pteropods, with their distribution (data from OBIS) overlaid on each map in purple dots. A) Pre-industrial aragonite saturation state, B) year 2020 aragonite saturation state, C) aragonite saturation state conditions at 10% reduction from pre-industrial levels, and D) aragonite saturation state conditions at 20% reduction from pre-industrial levels.

**
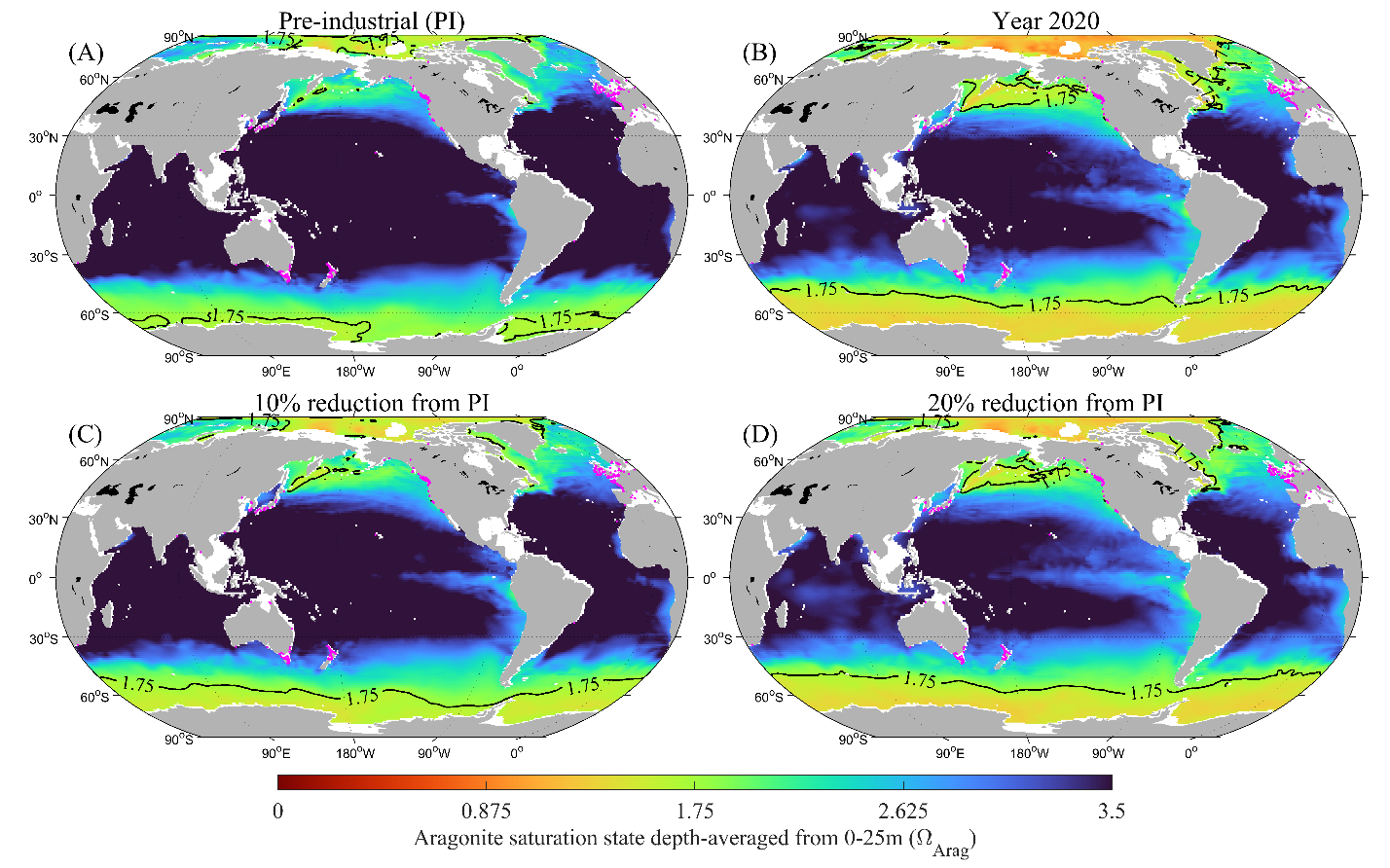
**

**Fig. S5**: Maps of surface ocean aragonite saturation state (Ω_Arag_), highlighting the 1.75 contour to show the regions that can be considered at zero relative production for Pacific oysters, with their distribution (data from OBIS) overlaid on each map in purple dots. A) Pre-industrial aragonite saturation state, B) year 2020 aragonite saturation state, C) aragonite saturation state conditions at 10% reduction from pre-industrial levels, and D) aragonite saturation state conditions at 20% reduction from pre-industrial levels.

**
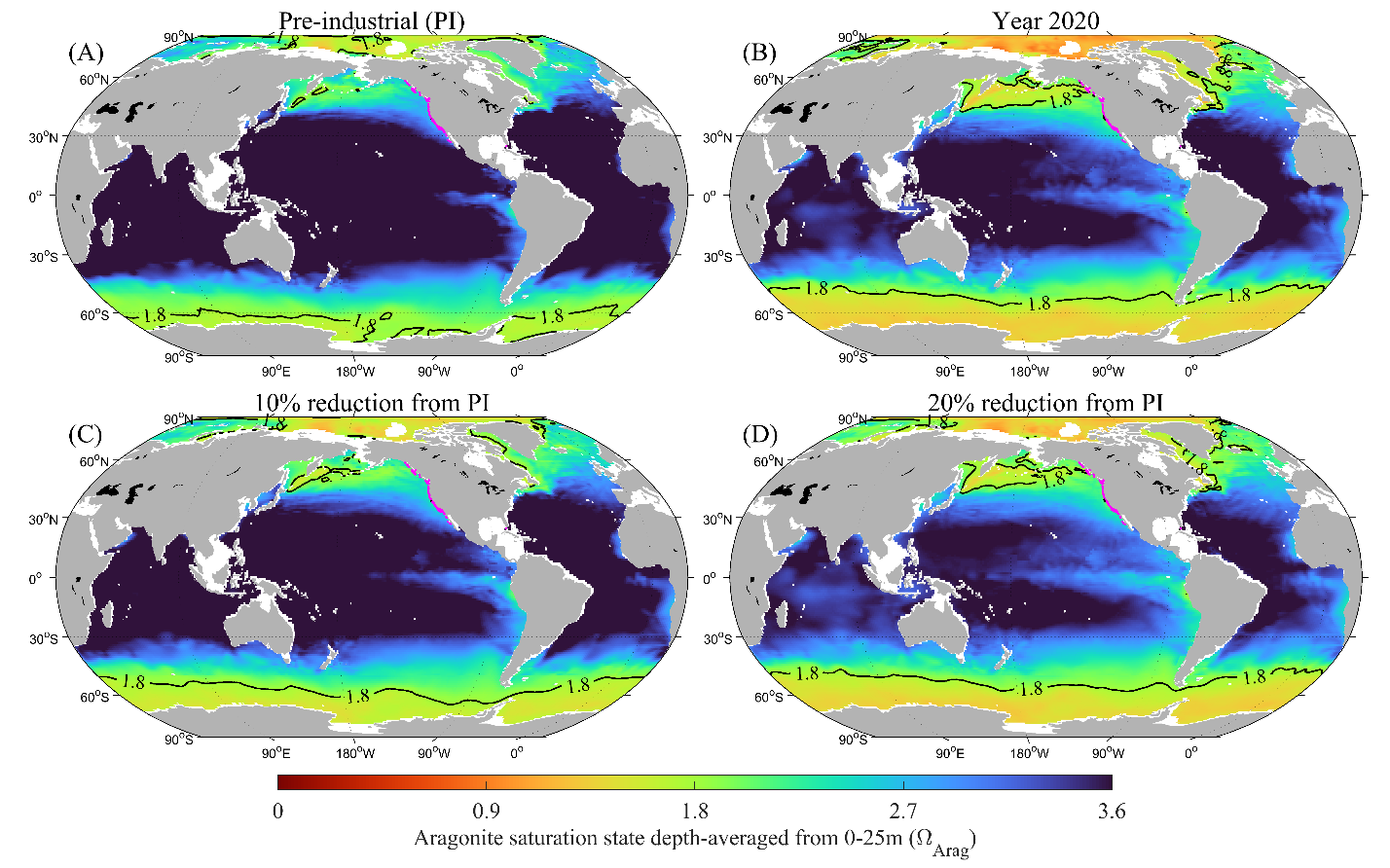
**

**Fig. S6**: Maps of surface ocean aragonite saturation state (Ω_Arag_), highlighting the 1.8 contour to show the regions that can be considered at risk for California mussel, with their distribution (data from OBIS) overlaid on each map in purple dots. A) Pre-industrial aragonite saturation state, B) year 2020 aragonite saturation state, C) aragonite saturation state conditions at 10% reduction from pre-industrial levels, and D) aragonite saturation state conditions at 20% reduction from pre-industrial levels.

**
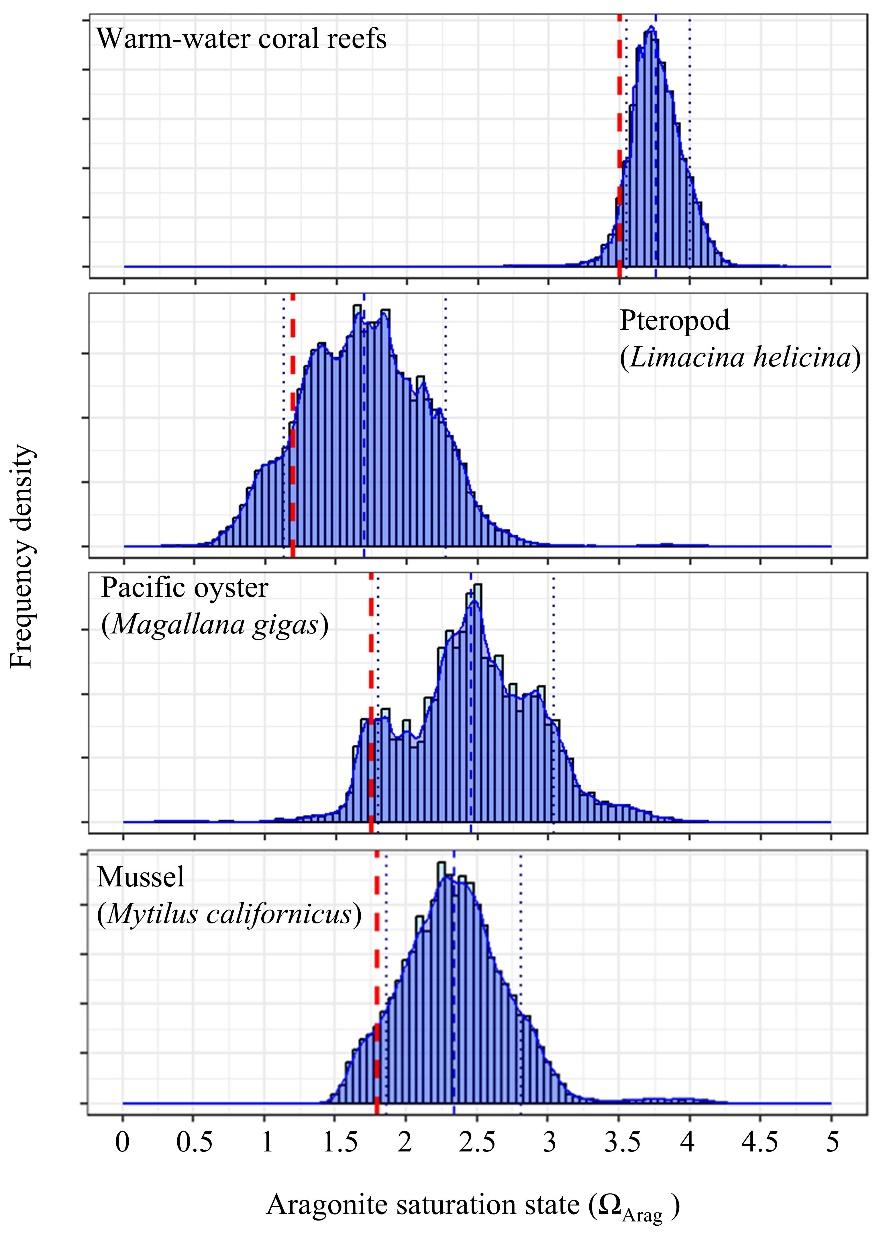
**

**Fig. S7**: Frequency density of aragonite saturation state from OceanSODA-ETHZv1 dataset, location-matched to occurrence data to describe the environmental envelopes for warm-water coral reefs, pteropods (*Limacina helicina*), bivalves – pacific oyster (*Magallana gigas*) and mussels (*Mytlius californicus*). Dashed blue line shows the median, dotted blue lines shows the 10^th^ centile and 90^th^ centile points, thick red dashed line shows the threshold value as found in the literature, usually from laboratory results (see methods): 3.5 for warm-water coral reefs, 1.2 for pteropods, 1.75 for pacific oyster and 1.8 for mussels.

**Table S1:** Aragonite saturation state (inter-model median, area-averaged) for the global ocean and regional ocean basins for each depth layer (0, 50, 100 and 200 m) for pre-industrial years 1750 and 1850, and present-day year 2020.

|  | Year 1750 | | | | Year 1850 | | | | Year 2020 | | | |
| --- | --- | --- | --- | --- | --- | --- | --- | --- | --- | --- | --- | --- |
|  | 0 m | 50 m | 100 m | 200 m | 0 m | 50 m | 100 m | 200 m | 0 m | 50 m | 100 m | 200 m |
| Global | 3.57 | 3.49 | 3.06 | 2.41 | 3.44 | 3.39 | 2.97 | 2.35 | 2.90 | 2.82 | 2.43 | 1.90 |
| Arctic | 2.06 | 1.92 | 1.8 | 1.84 | 1.97 | 1.84 | 1.74 | 1.78 | 1.49 | 1.41 | 1.33 | 1.44 |
| Pacific-N | 2.53 | 2.28 | 1.96 | 1.36 | 2.45 | 2.2 | 1.9 | 1.33 | 1.94 | 1.71 | 1.43 | 1.02 |
| Pacific-C | 4.09 | 4.02 | 3.53 | 2.63 | 2.91 | 3.92 | 3.44 | 2.56 | 3.36 | 3.30 | 2.85 | 2.06 |
| Atlantic-N | 3.00 | 2.85 | 2.66 | 2.51 | 3.99 | 2.76 | 2.58 | 2.44 | 2.36 | 2.24 | 2.08 | 1.99 |
| Atlantic-C | 4.23 | 4.08 | 3.53 | 2.80 | 4.13 | 3.97 | 3.44 | 2.73 | 3.49 | 3.35 | 2.86 | 2.25 |
| Indian | 4.20 | 4.06 | 3.34 | 2.60 | 4.10 | 3.96 | 3.26 | 2.54 | 3.45 | 3.31 | 2.65 | 2.03 |
| Southern | 2.30 | 2.35 | 2.14 | 1.87 | 2.23 | 2.27 | 2.07 | 1.82 | 1.77 | 1.82 | 1.65 | 1.48 |

**Table S2:** Inter-model standard deviations of aragonite saturation state (area-averaged) for the global ocean and regional ocean basins for each depth layer (0, 50, 100 and 200 m) for pre-industrial years 1750 and 1850, and present-day year 2020.

|  | Year 1750 | | | | Year 1850 | | | | Year 2020 | | | |
| --- | --- | --- | --- | --- | --- | --- | --- | --- | --- | --- | --- | --- |
|  | 0 m | 50 m | 100 m | 200 m | 0 m | 50 m | 100 m | 200 m | 0 m | 50 m | 100 m | 200 m |
| Global | 0.12 | 0.10 | 0.12 | 0.11 | 0.12 | 0.11 | 0.11 | 0.11 | 0.06 | 0.03 | 0.07 | 0.17 |
| Arctic | 0.31 | 0.21 | 0.18 | 0.16 | 0.30 | 0.2 | 0.17 | 0.15 | 0.14 | 0.09 | 0.08 | 0.08 |
| Pacific-N | 0.15 | 0.13 | 0.15 | 0.15 | 0.14 | 0.12 | 0.14 | 0.14 | 0.06 | 0.06 | 0.07 | 0.14 |
| Pacific-C | 0.13 | 0.13 | 0.14 | 0.13 | 0.16 | 0.13 | 0.13 | 0.12 | 0.07 | 0.08 | 0.12 | 0.26 |
| Atlantic-N | 0.17 | 0.16 | 0.14 | 0.12 | 0.12 | 0.16 | 0.14 | 0.12 | 0.08 | 0.08 | 0.08 | 0.07 |
| Atlantic-C | 0.10 | 0.10 | 0.10 | 0.13 | 0.10 | 0.09 | 0.10 | 0.12 | 0.04 | 0.06 | 0.12 | 0.22 |
| Indian | 0.10 | 0.10 | 0.12 | 0.09 | 0.09 | 0.10 | 0.11 | 0.08 | 0.05 | 0.07 | 0.12 | 0.23 |
| Southern | 0.08 | 0.08 | 0.08 | 0.09 | 0.08 | 0.08 | 0.08 | 0.08 | 0.04 | 0.04 | 0.05 | 0.05 |

**Table S3**: Area-averaged global surface aragonite saturation state (Ω_Arag_) for year 2020 from this study (see Tables S1 and S2), the OceanSODA-ETHZv1.2023 product (Gregor and Gruber, 2021) and the Copernicus CMEMS-LSCE product (Chau et al., 2024). For (Jiang et al., 2023) the uncertainty represents inter-model standard deviations. For OceanSODA-ETHZv1.2023 and CMEMS-LSCE the uncertainty represents propagated uncertainty from the calculations of aragonite saturation state (details in referenced papers).

| Product | Resolution | Ω_Arag_ |
| --- | --- | --- |
| 80% boundary (Richardson et al. 2023) |  | 2.75 |
| 80% boundary (this paper) |  | 2.80 ± 0.05 |
| Jiang et al. (2023) (this paper) | 1^°^x1^°^, decadal, global | 2.90 ± 0.06 |
| OceanSODA-ETHZv1.2023 | 1^°^x1^°^, monthly, global (to ice edge) | 2.64 ± 0.03 |
| CMEMS-LSCE | 1^°^x1^°^, monthly, global (75^°^S to 80^°^N) | 2.65 ± 0.16 |

**Table S4:** Pre-industrial temperature, salinity, total alkalinity (TA), taken from Jiang et al. (2023)

| **Region** | **Temperature (°C)** | **Salinity (psu)** | **TA (µmol kg^-1^)** |
| --- | --- | --- | --- |
| **Global** | 18.19 | 34.67 | 2294 |
| **Arctic** | 1.1 | 31.09 | 2151 |
| **Pacific-N** | 9.54 | 32.82 | 2213 |
| **Atlantic-N** | 11.42 | 34.84 | 2303 |
| **Pacific-C** | 24.02 | 34.9 | 2296 |
| **Atlantic-C** | 23.23 | 36.1 | 2365 |
| **Indian** | 24.86 | 35 | 2295 |
| **Southern** | 5.13 | 34.17 | 2291 |

Table S5: Individual dataset citations for *Magallana gigas* occurrence data, obtained from the Ocean Biodiversity Information Service mapper at [www.obis.org](http://www.obis.org) downloaded on 13^th^ November 2023. N/A = no information available.

| **Dataset name** | **Citation** | **Records** |
| --- | --- | --- |
| ICES contaminants and biological effects | ICES Contaminants and biological effects database (DOME – Biota). The International Council for the Exploration of the Sea, Copenhagen. 2010. Online source: http://ecosystemdata.ices.dk. | 871 |
| Index Site Surveys Data for Olympia Oysters, Ostrea lurida, in British Columbia – 2009 to 2017 | Bureau D (2021): Index Site Surveys Data for Olympia Oysters, Ostrea lurida, in British Columbia – 2009 to 2017. v1.4. Fisheries and Oceans Canada. Dataset/Samplingevent. http://ipt.iobis.org/obiscanada/resource?r=bc_index-site-surveys_olympia-oysters&v=1.4 | 492 |
| mabik_mo | N/A | 252 |
| eDNA data collected in Suva, Fiji by the PacMAN project | N/A | 242 |
| DFO Pacific Shorekeepers Intertidal Survey – a community based project | DFO (2013). DFO Pacific Shorekeepers Intertidal Survey – a community based project. Version 1 In OBIS Canada Collections. Bedford Institute of Oceanography, Dartmouth, NS, Canada. Published by OBIS. http://www.iobis.org/. | 212 |
| A Record of the Intertidal Malacofauna of Cape Bansho, Wakayama, Japan, from 1985 to 2010 | Ohgaki, S., K. Komemoto and N. Funayama (2011) A record of the intertidal malacofauna of Cape Bansho, Wakayama, Japan, from 1985 to 2010. Publications of the Seto Marine Biological Laboratory Special Publication Series, 11, 1-311. Dataset published in electronic format in 2020. | 194 |
| Marine Recorder Snapshot extract of surveys entered by Kent Wildlife Trust | Kent Wildlife Trust (2021): Marine Recorder Snapshot extract of surveys entered by Kent Wildlife Trust. v2.1. Marine Biological Association. Dataset/Samplingevent. https://doi.org/10.17031/rkwbds | 187 |
| 2014 Marine Biological Association (MBA) Yealm River non-native species timed search | MBA (2022) 2014 Marine Biological Association (MBA) Yealm River non-native species timed search | 167 |
| Survey data of tidal flats on the Monitoring sites 1000 project, BDCJ | Biodiversity Center of Japan, Ministry of the Environment. Data file of tidal flats survey on Monitoring sites 1000 project at http://www.biodic.go.jp/moni1000/findings/data/index_file_tidalflats.html. | 160 |
| Tasmanian Marine Molluscs Observational Records (2003-2017) | Grove, S.J. (2017). A Guide to the Seashells and other Marine Molluscs of Tasmania, web-site. http://www.molluscsoftasmania.org.au | 156 |
| The entire coast survey of Hatakejima Islands from 1983 | Nakano, T. (2021) The entire coast survey of Hatakejima Islands from 1983. Available at https://doi.org/10.48518/00011. Accessed on yyyy-mm-dd. | 121 |
| Marine Recorder Snapshot extract of surveys entered by National Museums Northern Ireland (NMNI) | National Museums Northern Ireland (NMNI) (2021): Marine Recorder Snapshot extract of surveys entered by National Museums Northern Ireland (NMNI). v2.0. Marine Biological Association. Dataset/Samplingevent. https://doi.org/10.17031/frdvov | 114 |
| Marine species citizen-science observations from NatureWatch NZ | NatureWatch NZ (2016). Marine species citizen-science observations from NatureWatch NZ. Southwestern Pacific OBIS, National Institute of Water and Atmospheric Research (NIWA), Wellington, New Zealand, 13030 records, Online http://nzobisipt.niwa.co.nz/resource.do?r=naturewatchnz released on March 26, 2017. | 113 |
| 2014 Marine Biological Association of the UK (MBA) Noss Mayo Yealm Estuary Transect Survey of Crassostrea gigas | MBA (2023) 2014 Marine Biological Association of the UK (MBA) Noss Mayo Yealm Estuary Transect Survey of Crassostrea gigas | 93 |
| 2014 Marine Biological Association of the UK (MBA) Noss Mayo Yealm Estuary Transect Survey of Crassostrea gigas | N/A | 93 |
| HELCOM/OSPAR Ballast water observations | HELCOM/OSPAR Joint Ballast Water Exemptions Decision Support Tool: https://maps.helcom.fi/website/RA_tool/ HELCOM and OSPAR, 2020 | 84 |
| Marine Life Survey Data (collected by volunteers) collated by MarLIN | Parr, J. Marine Life Survey Data (collected by volunteers) collated by MarLIN. MarLIN, collated Marine Life Survey Datasets, Marine Biological Association of the UK, Plymouth, UK. | 80 |
| 2001-2019 The Marine Biological Association of the UK (MBA) Shore Thing Survey | 45: Marine Biological Association of the UK (MBA) (2024): 2001-2019 The Marine Biological Association of the UK (MBA) Shore Thing Survey. v1.7. Marine Biological Association. Dataset/Samplingevent. 10.17031/64d60541c7e53 | 79 |
| Marine Recorder Snapshot extract of surveys entered by The archive for marine species and habitats data (DASSH) | The archive for marine species and habitats data (DASSH) (2021): Marine Recorder Snapshot extract of surveys entered by The archive for marine species and habitats data (DASSH). v2.1. Marine Biological Association. Dataset/Samplingevent. https://doi.org/10.17031/myrqac | 76 |
| 1915-2016 Department for Environment Food & Rural Affairs (Defra), Marine Strategy Framework Directive (MSFD) Collation of invasive non-indigenous species | Department for Environment Food and Rural Affairs (Defra) (2018): 1915-2016 Department for Environment Food & Rural Affairs (Defra), Marine Strategy Framework Directive (MSFD) Collation of invasive non-indigenous species. https://doi.org/10.17031/f0vfo3 | 67 |
| HELCOM/OSPAR Netherlands ports water sampling | HELCOM/OSPAR Joint Ballast Water Exemptions Decision Support Tool: https://maps.helcom.fi/website/RA_tool/ HELCOM and OSPAR, 2020 | 59 |
| Marine Recorder Snapshot extract of surveys entered by SeaSearch | SeaSearch (2021): Marine Recorder Snapshot extract of surveys entered by SeaSearch. v2.0. Marine Biological Association. Dataset/Samplingevent https://doi.org/10.17031/yq0gbg | 57 |
| Rocky shore macrobiota of southeastern Osaka Bay: Results of surveys | Association for the Research of Littoral Organisms in Osaka Bay (2024 onwards) Rocky shore macrobiota of southeastern Osaka Bay: Results of surveys at https://doi.org/10.48518/00025. | 56 |
| Marine Recorder Snapshot extract of surveys entered by Natural England | Natural England (NE) (2021): Marine Recorder Snapshot extract of surveys entered by Natural England. v2.0. Marine Biological Association. Dataset/Samplingevent. https://doi.org/10.17031/thn0xd | 53 |
| Tasmanian Museum and Art Gallery Invertebrate Collection - marine records | Byrne C (2023): Tasmanian Museum and Art Gallery Invertebrate Collection - marine records. v1.8. CSIRO National Collections and Marine Infrastructure (NCMI) Information and Data Centre (IDC). Dataset/Occurrence. https://www.marine.csiro.au/ipt/resource?r=ala_co198&v=1.8 | 51 |
| The south coast survey of Hatakejima Islands from 1969 | Nakano, T. (2021) The south coast survey of Hatakejima Islands from 1969. Available at https://doi.org/10.48518/00012. | 48 |
| Nonindigenous Aquatic Species (NAS) Database Non-freshwater Specimens | N/A | 39 |
| Tasmanian Museum and Art Gallery provider for OZCAM - marine records | Webmaster O (2022): Tasmanian Museum and Art Gallery provider for OZCAM - marine records. v1.9. CSIRO National Collections and Marine Infrastructure (NCMI) Information and Data Centre (IDC). Dataset/Occurrence. https://www.marine.csiro.au/ipt/resource?r=tmag_marine&v=1.9 | 34 |
| Macrophytes occurrence data within French Mediterranen Lagoon since 2006 | Institut Français de Recherche pour l’Exploitation de la MER (2022). Macrophytes occurrence data within French Mediterranen Lagoon since 2006 | 33 |
| Simultaneous surveys of littoral organisms in Osaka Bay in 2024 | Osaka Bay Environmental Restoration Liaison Committee (2024) Simultaneous surveys of littoral organisms in Osaka Bay in 2024. https://doi.org/10.48518/00028. | 26 |
| HELCOM/OSPAR Germany ports water sampling | HELCOM/OSPAR Joint Ballast Water Exemptions Decision Support Tool: https://maps.helcom.fi/website/RA_tool/ HELCOM and OSPAR, 2020 | 26 |
| Malacology Collection at the Academy of Natural Sciences of Philadelphia | N/A | 25 |
| Galiano Island BC Canada Marine Zoology 1893–2021 | Simon A, Basman A (2022): Galiano Island BC Canada Marine Zoology 1893–2021. v1.5. Biodiversity Data Journal. Dataset/Occurrence. https://ipt.pensoft.net/resource?r=galiano-data-paper-part-i&v=1.5 | 23 |
| Marine Invertebrata specimen database of Osaka Museum of Natutal History | Ishida S (2016). Marine Invertebrata specimen database of Osaka Museum of Natutal History. National Institute of Genetics, ROIS. Occurrence dataset https://doi.org/10.15468/zhubgk accessed via GBIF.org on yyyy-mm-dd. | 21 |
| SHARK - Regional monitoring and monitoring projects of Epibenthos in Sweden since 1994 | Swedish county administration boards, Swedish municipalities, Swedish coalitions of water conservation, Swedish Meteorological and Hydrological Institute, et al (2017). SHARK - Regional monitoring and monitoring projects of Epibenthos in Sweden since 1994 | 21 |
| Simultaneous surveys of littoral organisms in Osaka Bay | Osaka Bay Environmental Restoration Liaison Committee (2023) Simultaneous surveys of littoral organisms in Osaka Bay. https://doi.org/10.48518/00024. | 21 |
| Porcupine Marine Natural History Society Dataset | Porcupine Marine Natural History Society (2024): Porcupine Marine Natural History Society Dataset. v3.0. Marine Biological Association. Dataset/Samplingevent. https://doi.org/10.17031/9ok65b | 19 |
| Benthic Network | Agence pour la recherche et la Valorisation Marines (ARVAM); Cellule De Suivi Du Littoral Normand (CSLN); Creocean; Groupe d'Etude des Milieux Estuariens et Littoraux; Groupe d'étude des milieux estuariens et littoraux Normandie (GEMEL Normandie); Hemisphere Sub; Impact Mer; Institut Français de Recherche pour l'Exploitation de la Mer (IFREMER); MAREX Expertise & Conseil en Environnement Marin (MAREX); National Natural History Museum Paris; CRESCO - Station de biologie marine de Dinard; National Natural History Museum Paris; Station de Biologie Marine de Concarneau; Pierre & Marie Curie University; Roscoff biology station (RBS); TBM Environnement; The National Center for Scientific Research; Laboratoire d'Ecogéochimie des Environnements Benthiques UMR 8222 (LECOB); The National Center for Scientific Research; Laboratoire d’Océanologie et de Géosciences - UMR 8187 LOG (LOG); Université Bordeaux 1; Environnements et Paléoenvironnements Océaniques (EPOC); Université de Bretagne Occidentale; Institut Universitaire Européen de la Mer (IUEM); Université de Bretagne Occidentale; Laboratoire d'écophysiologie et de biotechnologie des halophytes et algues Marines (IUEM-LEBHAM); Université de Bretagne Occidentale; Laboratory of Sciences of the Marine Environment CNRS-UMR6539 (LEMAR); Université de la Réunion; Laboratoire d'Ecologie Marine (ECOMAR); Université de Liège; Station de Recherche Sous-marines et Océanographiques (STARESO); Université de Nantes; Laboratoire de Biologie Marine (Bio-littoral); Université des Sciences et Technologies de Lille; Station Marine de Wimereux (INSU/CNRS); (2019): REBENT - Réseau Benthique. | 19 |
| Queen Victoria Museum and Art Gallery - Marine Invertebrates | Reid C (2023): Queen Victoria Museum and Art Gallery - Marine Invertebrates. v1.8. CSIRO National Collections and Marine Infrastructure (NCMI) Information and Data Centre (IDC). Dataset/Occurrence. https://www.marine.csiro.au/ipt/resource?r=ala_co258&v=1.8 | 16 |
| Specific diversity data of macrobenthic communities in the "Rivière de Morlaix" study site in the English Channel from 1977 to 1996 | HOUBIN C, THIEBAUT É, HOEBEKE M (2019): Specific diversity data of macrobenthic communities in the "Rivière de Morlaix" study site in the English Channel from 1977 to 1996. https://doi.org/10.21411/qxef-sr30 | 15 |
| Marine Intertidal Phase 1 species dataset from the Countryside Council for Wales 1996-2005 | UK National Biodiversity Network, Countryside Council for Wales - Marine Intertidal Phase 1 species dataset from the Countryside Council for Wales 1996-2005. https://doi.org/10.15468/kflo7m | 15 |
| SHARK - National Epibenthos monitoring in Sweden since 1992 | Stockholm University, University of Gothenburg, Linnaeus University , Swedish Agency for Marine and Water Management, Swedish Environmental Protection Agency and Swedish Meteorological and Hydrological Institute (2017). SHARK - National Epibenthos monitoring in Sweden since 1992. | 13 |
| Marine biological observation data from coastal and offshore surveys around New Zealand | SWPRON (2014). Marine biological observation data from coastal and offshore surveys around New Zealand. Southwestern Pacific OBIS, National Institute of Water and Atmospheric Research (NIWA), Wellington, New Zealand, Online http://nzobisipt.niwa.co.nz/resource.do?r=mbis_nz | 13 |
| Explore Your Shore | National Biodiversity Data Centre (NBDC), Ireland; (2022): Explore Your Shore https://dx.doi.org/10.14284/563 | 13 |
| Marine Recorder Snapshot extract of surveys entered by JNCC | Joint Nature Conservation Committee (JNCC) (2021): Marine Recorder Snapshot extract of surveys entered by JNCC. v2.0. Marine Biological Association. Dataset/Samplingevent https://doi.org/10.17031/mehqrq | 11 |
| Delaware Museum of Nature and Science – Mollusks | N/A | 10 |
| Taxonomic Information System for the Belgian coastal area | Flanders Marine Institute (VLIZ). Taxonomic Information System for the Belgian coastal area. 10 Aug 2004, Oostende, Belgium. | 8 |
| KOBIS database | N/A | 8 |
| Museums Victoria Marine Invertebrates Collection | Jo Taylor J (2023): Museums Victoria Marine Invertebrates Collection. v1.12. CSIRO National Collections and Marine Infrastructure (NCMI) Information and Data Centre (IDC). Dataset/Occurrence. https://www.marine.csiro.au/ipt/resource?r=nmv_marine_inverts&v=1.12 | 8 |
| Museum and Art Gallery of the Northern Territory Malacology Collection - marine records | Dally G (2023): Museum and Art Gallery of the Northern Territory Malacology Collection - marine records. v1.11. CSIRO National Collections and Marine Infrastructure (NCMI) Information and Data Centre (IDC). Dataset/Occurrence. https://www.marine.csiro.au/ipt/resource?r=magnt_malacology&v=1.11 | 7 |
| Auckland Museum NZ Marine Collection | Blom W, Moriarty A (2018). Auckland Museum NZ Marine Collection. Version 1.11. Auckland War Memorial Museum. Occurrence Dataset https://doi.org/10.15468/plyefd accessed via GBIF.org on 2018-01-15. | 7 |
| DMNS Marine Invertebrate Collection (Arctos) | Denver Museum of Nature & Science Marine Invertebrate Collection | 7 |
| HELCOM/OSPAR Sweden ports water sampling | HELCOM/OSPAR Joint Ballast Water Exemptions Decision Support Tool: https://maps.helcom.fi/website/RA_tool/ HELCOM and OSPAR, 2020. | 6 |
| MarBEF Publication Series data | MarBEF (2006) MarBEF Publication Series data. Available online on EurOBIS. Consulted on dd-mm-yyyy. | 6 |
| Mollusc data of Molltax, Norway | Natural History Museum, University of Oslo, Mollusk data of Molltax, Norway https://doi.org/10.15468/vgbdvx | 6 |
| Dutch national shellfish monitoring in the coastal zone | Wageningen Marine Research (2019). WOT-schelpdieren: Dutch national shellfish monitoring in the coastal zone | 5 |
| SHARK - Regional monitoring, recipient control and monitoring projects of zoobenthos in Sweden since 1972 | Swedish county administration boards, Swedish municipalities, Swedish coalitions of water conservation, Swedish companies and Swedish Meteorological and Hydrological Institute et.al.(2021). Regional monitoring, recipient control and monitoring projects of zoobenthos in Sweden since 1972. https://doi.org/10.15468/cesssx | 5 |
| Demersal and mega-benthic species from the MEDITS (Mediterranean International Trawl Survey) project on the Spanish continental shelf between 1994 and 2009 | Massuti, Enric; García, Cristina; Guijarro, Beatriz; Quetglas, Antoni; Gil de Sola, Luis; Instituto Español de Oceanografía (IEO); Spain; (2017): Demersal and mega-benthic species from the MEDITS (Mediterranean International Bottom Trawl Survey) program at the Spanish continental shelf and upper slope between 1994 and 2009. https://dx.doi.org/10.14284/420 | 4 |
| Monitoring of macrozoobenthos in the Voordelta | Borst, J.C.;Stolte, W.; van der Kamp, P.; Rijkswaterstaat (RWS); Havenbedrijf Rotterdam N.V. (HbR); Deltares; Imares; (2014): Monitoring of macrozoobenthos in the Voordelta. | 4 |
| INRAM: Benthic fauna monitoring in Belgian harbours and Belgian coastal area | Marine Biology Section, Ugent. Belgium. INRAM. Benthic fauna monitoring- SSD - Belgian Science Policy. Available online http://www.vliz.be/projects/inram/imers.php. [accessed on ‘date’] | 4 |
| A Biotic Database of Indo-Pacific Marine Mollusks | Rosenberg et al. A Biotic Database of Indo-Pacific Marine Mollusks. Morris, P., Pennsylvania, USA. http://clade.acnatsci.org/obis/ | 4 |
| UF Invertebrate Zoology | N/A | 4 |
| Marine impactful cryptogenic and alien species in the Greek Seas: A georeferenced dataset (1893-2020) | Sini M, Ragkousis M, Koukourouvli N, Katsanevakis S, Zenetos A (2024). Marine impactful cryptogenic and alien species in the Greek Seas: A georeferenced dataset (1893-2020). Version 2.0. Hellenic Center for Marine Research. Occurrence dataset. https://doi.org/10.25607/t2smha | 3 |
| CAS Invertebrate Zoology (IZ) | N/A | 3 |
| PANGAEA - Data from various sources | N/A | 3 |
| Benthos Oosterschelde 1959-2015 | NIOZ (2023) Benthos Oosterschelde 1959-2015 | 3 |
| Intertidal and Subtidal Macroalgae taxa data from French REBENT Network | REBENT network (2019). Intertidal and Subtidal Macroalgae taxa data in ODV format from French REBENT Network. SEANOE. Https://doi.org/10.17882/60891 | 3 |
| ARMS-MBON data on long-term monitoring of hard-bottom communities: COI results from 2018-2020 | Obst, M.; Exter, K.M.; Pavloudi, C.; Pagnier, J.; Genin, A.; Balazy, P.; Broudin, C.; Cancio, I.; Chelchowski, M.; Chrismas, N.; Comtet, T.; Dailianis, T.; Daraghmeh, N.; Deneudt, K.; Diaz de Cerio, O.; González, J.; Kauppi, L.; Kristoffersen, J.B.; Kuklinski, P.; Lasota, R.; Lévêque, L.; Liraz, L.; Loisel, S.; Malachowicz, M.; Mavric, B.; Mortelmans, J.; Paredes Rosendo, E.; Pocwierz-Kotus, A.; Reiss, H.; Santi, I.; Solbakken, J.; Souza Troncoso, J.; Staehr, P.; Tajadura, J.; Thyrring, J.; Viard, F.; Zafeiropoulos, H.; Zbawicka, M.; University of Gothenburg, Faculty of Science, Department of Marine Sciences: Sweden; Flanders Marine Institute: Belgium; Hellenic Centre for Marine Research, Institute of Marine Biology, Biotechnology and Aquaculture: Greece; The Interuniversity Institute for Marine Sciences Of Eilat: Israel; Polish Academy of Sciences, Institute of Oceanology: Poland; Sorbonne Université, Station Biologique de Roscoff: France; University of the Basque country, Plentzia Marine Station: Spain; Marine Biological Association of the UK: United Kingdom; University of Vigo, Ecology and Animal Biology Department, Station of marine Science of Toralla: Spain; University of Helsinki, Tvärminne Zoological Station: Finland; University of Gdansk, Faculty of Biology, Geography and Oceanology, Institute of Oceanography: Poland; National Institute of Biology, Marine Biological Station Piran: Slovenia; Nord University, Faculty of Bioscience and Aquaculture: Norway; European Marine Biological Research Centre: France; University of Vigo, Ecology and Animal Biology Department, Faculty of Marine Sciences: Spain; University of Aarhus, Faculty of Technical Sciences, Department of Ecoscience: Denmark; (2020): ARMS-MBON data on long-term monitoring of hard-bottom communities: COI results from 2018-2020 https://dx.doi.org/10.14284/620 | 2 |
| NaGISA Project | N/A | 2 |
| ARMS-MBON data on long-term monitoring of hard-bottom communities: COI results from 2018-2020 | Obst, M.; Exter, K.M.; Pavloudi, C.; Pagnier, J.; Genin, A.; Balazy, P.; Broudin, C.; Cancio, I.; Chelchowski, M.; Chrismas, N.; Comtet, T.; Dailianis, T.; Daraghmeh, N.; Deneudt, K.; Diaz de Cerio, O.; González, J.; Kauppi, L.; Kristoffersen, J.B.; Kuklinski, P.; Lasota, R.; Lévêque, L.; Liraz, L.; Loisel, S.; Malachowicz, M.; Mavric, B.; Mortelmans, J.; Paredes Rosendo, E.; Pocwierz-Kotus, A.; Reiss, H.; Santi, I.; Solbakken, J.; Souza Troncoso, J.; Staehr, P.; Tajadura, J.; Thyrring, J.; Viard, F.; Zafeiropoulos, H.; Zbawicka, M.; University of Gothenburg, Faculty of Science, Department of Marine Sciences: Sweden; Flanders Marine Institute: Belgium; Hellenic Centre for Marine Research, Institute of Marine Biology, Biotechnology and Aquaculture: Greece; The Interuniversity Institute for Marine Sciences Of Eilat: Israel; Polish Academy of Sciences, Institute of Oceanology: Poland; Sorbonne Université, Station Biologique de Roscoff: France; University of the Basque country, Plentzia Marine Station: Spain; Marine Biological Association of the UK: United Kingdom; University of Vigo, Ecology and Animal Biology Department, Station of marine Science of Toralla: Spain; University of Helsinki, Tvärminne Zoological Station: Finland; University of Gdansk, Faculty of Biology, Geography and Oceanology, Institute of Oceanography: Poland; National Institute of Biology, Marine Biological Station Piran: Slovenia; Nord University, Faculty of Bioscience and Aquaculture: Norway; European Marine Biological Research Centre: France; University of Vigo, Ecology and Animal Biology Department, Faculty of Marine Sciences: Spain; University of Aarhus, Faculty of Technical Sciences, Department of Ecoscience: Denmark; (2020): ARMS-MBON data on long-term monitoring of hard-bottom communities: COI results from 2018-2020 https://dx.doi.org/10.14284/620 | 2 |
| 2018 Marine Biological Association of the UK (MBA) Yealm Bioblitz | 45: Marine Biological Association of the UK (MBA) (2024): 2018 Marine Biological Association of the UK (MBA) Yealm Bioblitz. v1.2. Marine Biological Association. Dataset/Samplingevent. 10.17031/64f9e88d81b0a | 2 |
| Australian Museum Malacology Collection - Marine records | Reid M (2022): Australian Museum Malacology Collection - Marine records. v1.10. CSIRO National Collections and Marine Infrastructure (NCMI) Information and Data Centre (IDC). Dataset/Occurrence. https://www.marine.csiro.au/ipt/resource?r=am_malacology&v=1.10 | 2 |
| 2009 - ongoing The Marine Biological Association of the UK (MBA) Devon and Cornwall Bioblitz Surveys | 45: Marine Biological Association of the United Kingdom (MBA) (2024): 2009 - ongoing The Marine Biological Association of the UK (MBA) Devon and Cornwall Bioblitz Surveys. v1.1. Marine Biological Association. Dataset/Samplingevent. 10.17031/64942873c2a44 | 2 |
| Marine flora and fauna records from the North-east Atlantic | Marine flora and fauna records from the North-east Atlantic. Porcupine Marine Natural History Society, UK - UK National Biodiversity Network. https://doi.org/10.15468/pcmg9q | 2 |
| Ocean Genome Legacy Collection | Accession ID OGL-#####. The Ocean Genome Legacy Center. Northeastern University. Published on the web at ogl.northeastern.edu/catalog. | 2 |
| 2017-2018 North Devon Coast Area of Outstanding Natural Beauty (AONB) North Devon Species Census | North Devon Coast Area of Outstanding Natural Beauty (AONB) (2021): 2017-2018 North Devon Coast Area of Outstanding Natural Beauty (AONB) North Devon Species Census. v2.0. Marine Biological Association. https://doi.org/10.17031/y7r3y3 | 2 |
| Epibenthos and demersal fish monitoring in function of dredge disposal monitoring in the Belgian part of the North Sea | Bio-environmental research group; Institute of Agricultural and Fisheries research (ILVO), Belgium; (2015): Epibenthos and demersal fish monitoring in function of dredge disposal monitoring in the Belgian part of the North Sea. https://dx.doi.org/10.14284/198 | 2 |
| Marine Recorder Snapshot extract of surveys entered by NatureScot | NatureScot (2021): Marine Recorder Snapshot extract of surveys entered by NatureScot. v2.0. Marine Biological Association. https://doi.org/10.17031/pqhlyg | 1 |
| Mollusca fauna from the Mediterranean reef ecosystem (1170 habitat) | Dimitris Poursanidis & Drosos Koutsoubas (2015). Mollusca fauna from the Mediterranean reef ecosystem (1170) – the zone of the photophilic algae. | 1 |
| Benthos monitoring in the intertidal mudflats of Pertuis-Charentais (Bay of Biscay) from 2004 on | Philippe, Anne S., Christine Plumejeaud-Perreau, Jérôme Jourde, Philippe Pineau, Nicolas Lachaussée, Emmanuel Joyeux, Frédéric Corre, Philippe Delaporte, Bocher Pierrick; (2016): Benthos monitoring in the intertidal mudflats of Pertuis-Charentais (Bay of Biscay) from 2004 on. https://dx.doi.org/10.14284/247 | 1 |
| Ecological hyperbenthic data of the Scheldt estuary: ENDIS-RISKS data (2002-2005) | N/A | 1 |
| Natal Museum - Mollusc Collection | Natal Museum - Mollusc Collection | 1 |
| NIWA Invertebrate Collection | NIWA (2018): NIWA Invertebrate Collection. v1.1. The National Institute of Water and Atmospheric Research (NIWA). Dataset/Occurrence. https://nzobisipt.niwa.co.nz/resource?r=obisspecify&v=1.1 | 1 |
| LBMRev | Aleffi, I. F., 2004: LBMRev dataset. Marine Biology Laboratory, University of Trieste, Italy | 1 |
| 2011 Marine Biological Association of the UK (MBA) Bovisand Surveying Habitats and Researching Coasts (SHARC) group and MBA members Alien Invaders timed survey | N/A | 1 |
| Benthic invertebrates abundance data from French REBENT network | REBENT network (2019). Benthic invertebrates abundance data in ODV format from French REBENT network. SEANOE. https://doi.org/10.17882/60681 | 1 |
| Roscoff Inventories: marine fauna and flora since 1800 | Houbin, C; Hoebeke, M (2019): Roscoff inventories: marine fauna and flora since 1800 https://doi.org/10.21411/qhtc-a855 | 1 |
| Marine Recorder Snapshot extract of surveys entered by Sussex Wildlife Trust | Sussex Wildlife Trust (2021): Marine Recorder Snapshot extract of surveys entered by Sussex Wildlife Trust. v2.1. Marine Biological Association. https://doi.org/10.17031/cyjwxu | 1 |
| Diveboard - Scuba diving citizen science observations | Diveboard - Scuba diving citizen science observations. Online at http://www.diveboard.com and http://ipt.diveboard.com/resource.do?r=diveboard-occurrences. https://dx.doi.org/10.15468/tnjrgy | 1 |
| Queensland Museum Molluscs - Marine records | Healy J (2022): Queensland Museum Molluscs - Marine records. v1.9. CSIRO National Collections and Marine Infrastructure (NCMI) Information and Data Centre (IDC). https://www.marine.csiro.au/ipt/resource?r=qm_molluscs&v=1.9 | 1 |
| Marine benthic animal data along Yokohama City Coast | Yokohama Environmental Science Research Institute (2023) Marine benthic animal data along Yokohama City Coast. Available at https://doi.org/10.48518/00023. | 1 |
| National Museum of Natural History Invertebrate Zoology Collections | National Museum of Natural History, Smithsonian Institution NMNH Invertebrate Zoology Collection Database. National Museum of Natural History, Smithsonian Institution, 10th and Constitution Ave. N.W., Washington, DC 20560-0193, 2001, Version 3.2.04 (0802221). | 1 |
| Biodiversity of the North Sea - Helgoland | GEO-Tag der Artenvielfalt, Artenvielfalt der Nordsee - Helgoland (accessed through GBIF data portal, http://data.gbif.org/datasets/resource/2688, yyyy-mm-dd) https://doi.org/10.15468/omx28y | 1 |
| Los Angeles Urban Ocean Expedition 2019 | N/A | 1 |
| Mollusca Collection - Museu Nacional/UFRJ | Pimenta A (2024). Mollusca Collection - Museu Nacional/UFRJ. Version 1.10. Museu Nacional / UFRJ. Occurrence dataset. https://ipt.sibbr.gov.br/mnrj/resource?r=mnrj_mollusca&v=1.10 | 1 |
| Marine species recorded in Ireland during field surveys by EcoServe, Ecological Consultancy Services Ltd. | Allen D., Beckett B., Brophy J., Costello M.J., Emblow C., Maciejewska B., McCrea M., Nash R., Penk M. & Tierney A. (2009) Marine species recorded in Ireland during field surveys by EcoServe, Ecological Consultancy Services Ltd. https://dx.doi.org/10.14284/485 | 1 |
| RSMP Baseline Dataset | Cooper et al. (2017). RSMP Baseline Dataset. Cefas, UK. V1. https://doi.org/10.14466/CefasDataHub.34 | 1 |
| Biodiversity of the North Sea - Sylt | GEO-Tag der Artenvielfalt, Artenvielfalt der Nordsee - Sylt (accessed through GBIF data portal, http://data.gbif.org/datasets/resource/2839, yyyy-mm-dd) https://doi.org/10.15468/nvhjkx | 1 |
| Norman and Florence Hammond records. Seawatch and coastal survey records | Norman and Florence Hammond records. Seawatch and coastal survey records. Cumbria Biodiversity Data Centre, UK - UK National Biodiversity Network. https://doi.org/10.15468/1u5tii | 1 |
| 2009 The Marine Biological Association of the UK (MBA) Wembury Bioblitz Survey | Lear D (2024): 2009 The Marine Biological Association of the UK (MBA) Wembury Bioblitz Survey. v3.1. Marine Biological Association. Dataset/Samplingevent. https://doi.org/10.17031/ppacwa | 1 |
| Epibenthos and demersal fish monitoring at long-term monitoring stations in the Belgian part of the North Sea | Bio-environmental research group; Institute of Agricultural and Fisheries research (ILVO), Belgium; (2015): Epibenthos and demersal fish monitoring at long-term monitoring stations in the Belgian part of the North Sea https://dx.doi.org/10.14284/54 | 1 |
| Gwaii Haanas Invertebrates | Sloan, N.A., Bartier, P.M., Austin, W.C. 2004. Gwaii Haanas Invertebrates (Living marine legacy of Gwaii Haanas II: Marine invertebrate baseline to 2000). Parks Canada-Technical Reports in Ecosystem Science. OBIS Canada, Bedford Institute of Oceanography, Dartmouth, Nova Scotia, Canada, Version 1, Digital, retrieved from http://iobis.org/. | 1 |
| Royal Belgian Institute of Natural Sciences Mollusca Collection (Mollusca) | Samyn, Y., Semal, P. (2020) Royal Belgian Institute of Natural Sciences Mollusca collection | 1 |
| Benthos Westerschelde 1963-2012 | NIOZ (2023) Benthos Westerschelde 1963-2012 | 1 |

Table S6: Individual dataset citations for *Mytilus californianicus* occurrence data, obtained from the Ocean Biodiversity Information Service mapper at [www.obis.org](http://www.obis.org) downloaded on 13^th^ November 2023. N/A = no information available.

| **Dataset name** | **Citation** | **Records** |
| --- | --- | --- |
| MARINe/PISCO: Intertidal: MARINe Long-Term Monitoring Surveys: Photo Plots and Transects Summarized | Multi-Agency Rocky Intertidal Network (MARINe), Partnership Interdisciplinary Studies Coastal Oceans for of (PISCO), Pete Raimondi, Richard Ambrose, Jack Engle, Jennifer Burnaford, Jayson Smith, Jenn Caselle, Jenny Waddell, Megan Dethier, Darren Fong, Ben Becker, Steven Fradkin, Heath Bohlmann, Melissa Miner, Jessica Curran, Lauren Pandori, Stephen Whitaker, & Jan Roletto. (2024). MARINe/PISCO: Intertidal: MARINe Long-Term Monitoring Surveys: Photo Plots and Transects Summarized. PISCO MN. doi:10.6085/AA/marine_ltm.4.14. | 3762 |
| MARINe/PISCO: Intertidal: MARINe Coastal Biodiversity Surveys: Point Contact Surveys Summarized | Multi-Agency Rocky Intertidal Network (MARINe), Partnership Interdisciplinary Studies Coastal Oceans for of (PISCO), & Pete Raimondi. (2023). MARINe/PISCO: Intertidal: MARINe Coastal Biodiversity Surveys: Point Contact Surveys Summarized. PISCO MN. doi:10.6085/AA/marine_cbs.5.6. | 328 |
| Gwaii Haanas Invertebrates | Sloan, N.A., Bartier, P.M., Austin, W.C. 2004. Gwaii Haanas Invertebrates (Living marine legacy of Gwaii Haanas II: Marine invertebrate baseline to 2000). Parks Canada-Technical Reports in Ecosystem Science. OBIS Canada, Bedford Institute of Oceanography, Dartmouth, Nova Scotia, Canada, Version 1, Digital, retrieved from http://iobis.org/. | 97 |
| DFO Pacific Shorekeepers Intertidal Survey – a community based project | DFO (2013). DFO Pacific Shorekeepers Intertidal Survey – a community based project. Version 1 In OBIS Canada Collections. Bedford Institute of Oceanography, Dartmouth, NS, Canada. Published by OBIS. http://www.iobis.org/. (consulted on [date]) | 45 |
| Delaware Museum of Nature and Science – Mollusks | N/A | 42 |
| Malacology Collection at the Academy of Natural Sciences of Philadelphia | N/A | 22 |
| National Museum of Natural History Invertebrate Zoology Collections | National Museum of Natural History, Smithsonian Institution NMNH Invertebrate Zoology Collection Database. National Museum of Natural History, Smithsonian Institution, 10th and Constitution Ave. N.W., Washington, DC 20560-0193, 2001, Version 3.2.04 (0802221). | 19 |
| UF Invertebrate Zoology | N/A | 16 |
| Ocean Genome Legacy Collection | Accession ID OGL-#####. The Ocean Genome Legacy Center. Northeastern University. Published on the web at ogl.northeastern.edu/catalog. | 10 |
| CAS Invertebrate Zoology (IZ) | N/A | 10 |
| BOLD Marine Invertebrate Data | N/A | 10 |
| DMNS Marine Invertebrate Collection (Arctos) | Denver Museum of Nature & Science Marine Invertebrate Collection | 4 |
| Metabarcoding of Benthic Marine Communities in Nova Scotia and New Brunswick | Krumhansl K, Brooks C, Kingsbury S, DiBacco C (2025). Metabarcoding of Benthic Marine Communities in Nova Scotia and New Brunswick. Version 1.1. Fisheries and Oceans Canada. Occurrence dataset. https://ipt.iobis.org/obiscanada/resource?r=metabarcoding_benthic_marine_communities_in_novascotia_newbrunswick_2021&v=1.1 | 2 |
| EPA'S EMAP Database | N/A | 1 |

Table S7: Individual dataset citations for *Limacina helicina* occurrence data, obtained from the Ocean Biodiversity Information Service mapper at [www.obis.org](http://www.obis.org) downloaded on 13^th^ November 2023. N/A = no information available.

| **Dataset name** | **Citation** | **Records** |
| --- | --- | --- |
| World Ocean Database 2009 | Baranova, O.K, T.D. O'Brien, T.P. Boyer and I.V. Smolyar (2009). Plankton data. Chapter 16 in Boyer, T. P., J. I. Antonov , O. K. Baranova, H. E. Garcia, D. R. Johnson, R. A. Locarnini, A. V. Mishonov, T. D. O'Brien, D. Seidov, I. V. Smolyar, M. M. Zweng, 2009. World Ocean Database 2009. S. Levitus, Ed., NOAA Atlas NESDIS 66, U.S. Gov. Printing Office, Wash., D.C., 216 pp., DVDs | 1586 |
| Puget Sound Zooplankton Monitoring Program (Salish Sea, USA), starting in 2014 | Keister J E, Winans A, Herrmann B, Kalata O, Mayorga E (2024). Puget Sound Zooplankton Monitoring Program (Salish Sea, USA), starting in 2014. Version 1.1. United States Geological Survey. Samplingevent dataset. https://doi.org/10.15468/e92gvx | 1441 |
| Zooplankton data from central and northern Strait of Georgia | Galbraith M (2021): Zooplankton data from central and northern Strait of Georgia. v1.4. Fisheries and Oceans Canada. http://ipt.iobis.org/obiscanada/resource?r=zoopl_sofg&v=1.4 | 1057 |
| DFO Pacific IOS zooplankton database - Line P | Galbraith, Moira. (2013) DFO Pacific IOS zooplankton database - Line P. Version 1 In OBIS Canada Digital Collections. Bedford Institute of Oceanography, Dartmouth, NS, Canada. Published by OBIS, Digital http://www.iobis.org/. Accessed on –INSERT DATE | 1045 |
| BioChem: Sameoto zooplankton collection | Sameoto, D.D., Kennedy, M., Spry, J.S, Spry, J.M. (2013). Zooplankton datasets collected using the BIONESS sampler, ring nets and an Icelandic high speed sampler, 1967-2006. OBIS Canada Digital Collections. Published by OBIS http://www.iobis.org/. Accessed on –INSERT DATE | 820 |
| BioChem: Atlantic Zone Monitoring Program (AZMP) Maritimes Region zooplankton collection | Kennedy, M.K., Spry, J.A. 2011. BioChem: Atlantic Zone Monitoring Program (AZMP) Maritimes Region zooplankton collection. OBIS Canada Digital Collections. OBIS Canada, Bedford Institute of Oceanography, Dartmouth, Nova Scotia, Canada, Version 1, Digital, retrieved from http://iobis.org/. | 740 |
| Archives of the Arctic Seas Zooplankton | Markhaseva, E.L., Golikov, A.A., Agapova, T.A., Beig, A.A. 1985 Archives of the Arctic Seas Zooplankton 1 | 419 |
| DFO Pacific IOS zooplankton database - Zooplankton samples collected during cruises to the Canadian Arctic, 2006-2009 | Nelson, John. (2014). DFO Pacific IOS zooplankton database - Zooplankton samples collected during cruises to the Canadian Arctic, 2006-2009. Version 1 In OBIS Canada Digital Collections. Bedford Institute of Oceanography, Dartmouth, NS, Canada. Published by OBIS, Digital http://www.iobis.org/. Accessed on –INSERT DATE | 377 |
| Zooplankton NOGAP32b 1986 | Hopky, G.E. Lawrence, M.J. and Chiperzak, D.B.; Central and Arctic Region, Department of Fisheries and Oceans, Winnipeg, Manitoba R3T 2N6. (2004) NOGAP B2; Zooplankton Data from the Canadian Beaufort Sea Shelf, 1986. Canadian Data Report of Fisheries and Aquatic Sciences: 923 | 253 |
| IMR Zooplankton Norwegian Sea | Broms, C. (2014). IMR Zooplankton Norwegian Sea. Institute of Marine Research, Norway | 199 |
| ZooplanktonBeaufortSeaNOGAP2 | Hopky, G.E. Lawrence, M.J. and Chiperzak, D.B.; Central and Arctic Region, Department of Fisheries and Oceans, Winnipeg, Manitoba R3T 2N6. (2004) NOGAP B2; Zooplankton Data from the Canadian Beaufort Sea Shelf, 1986. Canadian Data Report of Fisheries and Aquatic Sciences: 923 | 184 |
| ZooplanktonBeaufortSeaNOGAP1 | Hopky, G.E. Lawrence, M.J. and Chiperzak, D.B.; Central and Arctic Region, Department of Fisheries and Oceans, Winnipeg, Manitoba R3T 2N6. (1994) NOGAP B2; Zooplankton Data from the Canadian Beaufort Sea Shelf, 1984 and 1985. Canadian Data Report of Fisheries and Aquatic Sciences: 922. | 183 |
| BioChem: Atlantic Zone Monitoring Program (AZMP) Quebec Region zooplankton collection | Kennedy, M.K., St-Pierre., I. 2011. BioChem: Atlantic Zone Monitoring Program (AZMP) Quebec Region zooplankton collection. OBIS Canada Digital Collections. OBIS Canada, Bedford Institute of Oceanography, Dartmouth, Nova Scotia, Canada, Version 1, Digital, retrieved from http://iobis.org/. | 150 |
| IMR Zooplankton Barents Sea | Knutsen, T.; Dalpadado, P. (2014). IMR Zooplankton Barents Sea. Institute of Marine Research, Norway | 140 |
| WhiteSeaPlankton | N/A | 122 |
| Bongo Zooplankton Data from the R/V TINRO, NOAA Bell M. Shimada, F/V Northwest Explorer and R/V CCGS Sir John Franklin during the 2022 International Year of the Salmon Pan-Pacific Winter High Seas Expedition | Breckenridge, J., Galbraith, M., King, J., Pinchuk, A., Stark, C., & Pakhomov, E. (2023). Bongo Zooplankton Data from the R/V TINRO, NOAA Bell M. Shimada, F/V Northwest Explorer and R/V CCGS Sir John Franklin during the 2022 International Year of the Salmon Pan-Pacific Winter High Seas Expedition (v1.1). North Pacific Anadromous Fish Commission. https://doi.org/10.21966/ymv6-8024 | 117 |
| Zooplankton Bongo Net Data from the 2019 and 2020 Gulf of Alaska International Year of the Salmon Expeditions | Hunt, B., Mahara, N., & Pakhomov, E. (2023). Zooplankton Bongo Net Data from the 2019 and 2020 Gulf of Alaska International Year of the Salmon Expeditions (v1.0) [Data set]. North Pacific Anadromous Fish Commission. https://doi.org/10.21966/7cmt-ca72 | 95 |
| Zooplankton Species Biomass and Abundance Data, Arctic Marine Biodiversity Observing Network (AMBON) Chukchi Sea research cruise on the Norseman II, 11 August - 3 September 2015 | N/A | 88 |
| Structure and function of contemporary food webs on Arctic shelves: A panarctic comparison. The pelagic system of the Kara Sea- communities and components of carbon flow | H.J. Hirche, K.N. Kosobokova, B. Gaye-Haake, I. Harms Originator: B. Meon and E.-M. Nöthig (2006). Structure and function of contemporary food webs on Arctic shelves: A panarctic comparison. The pelagic system of the Kara Sea-communities and components of carbon flow | 87 |
| Zooplankton Species Distribution and Abundance Data, Arctic Marine Biodiversity Observing Network (AMBON) Chukchi Sea research cruise, August 2017 | N/A | 82 |
| BioChem: Davis Strait and Baffin Bay Zooplankton | Lewis, M.K. and D. Sameoto. (2017). BioChem: Davis Strait and Baffin Bay Zooplankton. Version 2 In OBIS Canada Digital Collections. Bedford Institute of Oceanography, Dartmouth, NS, Canada. Published by OBIS, Digital http://www.iobis.org/. | 75 |
| RMT Trawl catch from the 1992/93 V6 KROCK voyage | Australian Antarctic Data Centre. RMT Trawl catch from the 1992/93 V6 KROCK voyage. | 72 |
| Juday Net Zooplankton Data from the 2019 Gulf of Alaska International Year of the Salmon Expedition | Slabinsky, A., Somov, A., & Pakhomov, E. (2022). Juday Net Zooplankton Data from the 2019 Gulf of Alaska International Year of the Salmon Expedition (Version 1.0) [Data set]. North Pacific Anadromous Fish Commission. https://doi.org/10.21966/ZW3V-MD11 | 71 |
| Zooplankton densities collected from a seasonally hypoxic fjord (Hood Canal, Salish Sea, USA) on 2012-2013 cruises | Keister J E, Essington T, Horne J K, Parker-Stetter S, Herrmann B, Li L, Mayorga E, Winans A (2024). Zooplankton densities collected from a seasonally hypoxic fjord (Hood Canal, Salish Sea, USA) on 2012-2013 cruises. Version 1.2. United States Geological Survey. Samplingevent dataset. https://doi.org/10.15468/a7upu6 | 65 |
| Composition and distribution of the biomass of zooplankton in the central Arctic Basin 1975, 1976, 1977 | K.N. Kosobokova (1982) Composition and distribution of the biomass of zooplankton in the central Arctic Basin 1975, 1976, 1977 | 61 |
| The pre-winter 2007 vertical distribution of zooplankton in the Cape Bathurst and North Water polynyas, and Lancaster Sound, Canadian Arctic | N/A | 59 |
| DFO Pacific IOS zooplankton database - Zooplankton samples collected during cruises to the Canadian Arctic between 1987 and 2000 | Galbraith, M. (2013) DFO Pacific IOS zooplankton database - Zooplankton collection from Arctic cruises, 1987-2000. Version 1 In OBIS Canada Digital Collections. Bedford Institute of Oceanography, Dartmouth, NS, Canada. Published by OBIS, Digital http://www.iobis.org/. | 58 |
| Gwaii Haanas Invertebrates | Sloan, N.A., Bartier, P.M., Austin, W.C. 2004. Gwaii Haanas Invertebrates (Living marine legacy of Gwaii Haanas II: Marine invertebrate baseline to 2000). Parks Canada-Technical Reports in Ecosystem Science. OBIS Canada, Bedford Institute of Oceanography, Dartmouth, Nova Scotia, Canada, Version 1, Digital, retrieved from http://iobis.org/. | 53 |
| CASES2003_2004 | Fortier, L. Darnis, G. (2006) Vertical distribution of arctic mesozooplankton in Franklin Bay, southern Beaufort Sea, during winter 2003-04 | 46 |
| RMT Trawl catch from the 1990/91 V6 AAMBER2 voyage | Australian Antarctic Data Centre. RMT Trawl catch from the 1990/91 V6 AAMBER2 voyage. | 43 |
| Biological observations from the Discovery Investigations 1925-1952 | Southwestern Pacific OBIS (2014). Biological observations from the Discovery Investigations 1925-1935. Southwestern Pacific OBIS, National Institute of Water and Atmospheric Research (NIWA), Wellington, New Zealand, 33337 records, Online http://nzobisipt.niwa.co.nz/resource.do?r=discovery_reports released on January 23, 2015. | 41 |
| CASES: The pre-winter assemblages of southeastern Beaufort Sea | Darnis, Gérald, Barber, David G., and Fortier, Louis. 2015. CASES: The pre-winter assemblages of southeastern Beaufort Sea. Version 1 In ArcOD Digital Collections. Institute of Marine Science University of Alaska, Fairbanks, AK, USA. Published by OBIS, Digital http://www.iobis.org/. | 41 |
| Juday Net Zooplankton Data from the 2020 Gulf of Alaska International Year of the Salmon Expedition | Kuznetsova, N., Somov, A., & Pakhomov, E. (2022). Juday Net Zooplankton Data from the 2020 Gulf of Alaska International Year of the Salmon Expedition (Version 1.0) [Data set]. North Pacific Anadromous Fish Commission. https://doi.org/10.21966/G9WR-VS33 | 37 |
| RMT Trawl catch from the 1984/85 V5 SIBEX2 voyage | Australian Antarctic Data Centre. RMT Trawl catch from the 1984/85 V5 SIBEX2 voyage. | 35 |
| RMT Trawl catch from the 1982/83 V2 ADBEX I voyage | Australian Antarctic Data Centre. RMT Trawl catch from the 1982/83 V2 ADBEX I voyage. | 34 |
| The Ecology of the Inshore Marine Zooplankton of the Chukchi Sea near Point Barrow, Alaska | Douglas R. Redburn (1974) The Ecology of the Inshore Marine Zooplankton of the Chukchi Sea near Point Barrow, Alaska | 32 |
| BioChem: Zooplankton collected from Davis Strait, 1976-1977 | Spry, J.M. 2012. BioChem: Zooplankton collected from Davis Strait, 1976-1977. OBIS Canada Digital Collections. OBIS Canada, Bedford Institute of Oceanography, Dartmouth, Nova Scotia, Canada, Version 1, Digital, retrieved from http://iobis.org/. | 32 |
| Marine Biological Sample Database, JAMSTEC | Japan Agency for Marine-Earth Science and Technology (2016 onwards). JAMSTEC Marine Biological Samples Database. https://doi.org/10.48518/00001. | 30 |
| Barents Sea Zooplankton – March and May 1998 and July 1999. Day/night stratified zooplankton sampling onboard the Norwegian R/V Jan Mayen. Program ALV | N/A | 29 |
| Zooplankton Bering Strait Tiglax 1991 | N/A | 28 |
| Zooplankton abundance in the Kerguelen Axis region 2016 | Swadling, K. (2017, updated 2018) Zooplankton abundance in the Kerguelen Axis region | 27 |
| British Antarctic (Terra Nova) Expedition, 1910-1913 | Southwestern Pacific OBIS (2014). British Antarctic (Terra Nova) Expedition, 1910-1913. Southwestern Pacific OBIS, National Institute of Water and Atmospheric Research (NIWA), Wellington, New Zealand, 1779 records, Online http://nzobisipt.niwa.co.nz/resource.do?r=terranova released on July 29, 2014 | 25 |
| Metabarcoding of Benthic Marine Communities in Nova Scotia and New Brunswick | Krumhansl K, Brooks C, Kingsbury S, DiBacco C (2025). Metabarcoding of Benthic Marine Communities in Nova Scotia and New Brunswick. Version 1.1. Fisheries and Oceans Canada. Occurrence dataset. https://ipt.iobis.org/obiscanada/resource?r=metabarcoding_benthic_marine_communities_in_novascotia_newbrunswick_2021&v=1.1 | 24 |
| Seasonal dynamics of sub-ice fauna below pack ice in the Arctic (Fram Strait) | N/A | 22 |
| Biogeographic data from BODC - British Oceanographic Data Centre | British Oceanographic Data Centre, UK. Biogeographic data from BODC. in : EurOBIS. http://www.marbef.org/data/eurobissearch.php?dataprovider=47, accessed on [date]. | 22 |
| Oceanexploration2002_vers4 | R.R. Hopcroft, C. Clarke, R.J. Nelson, K.A. Raskoff. Zooplankton Communities of the Arctic�s Canada Basin: the contribution by smaller taxa. Polar Biology, 28: 198-206 | 21 |
| CAS Invertebrate Zoology (IZ) | N/A | 20 |
| Zooplankton and Ichthyoplankton Data Collected from the Chukchi and Beaufort Seas during the R/V Mirai Cruise, September 2002 | W. Walkusz, J.E Paulic, M.H Papst, S Kwasniewski, S. Chiba, and R.E Crawford (2008). Zooplankton and Ichthyoplankton Data Collected from the Chukchi and Beaufort Seas during the R/V Mirai Cruise, September 2002. Canadian Data Report of Fisheries and Aquatic Science (1211) | 20 |
| DFO-SABS: Plankton data collected during 1923 Strait of Belle Isle Expedition. | DFO. (2014). Entomostraca collected in and around the Strait of Belle isle during expeditions in 1923. Version 1 In OBIS Canada Digital Collections. Bedford Institute of Oceanography, Dartmouth, NS, Canada. Published by OBIS. http://www.iobis.org/. | 19 |
| RMT Trawl catch from the 1980/81 V5 FIBEX voyage | N/A | 19 |
| IMR Zooplankton North Sea | Falkenhaug, T. (2014). IMR Zooplankton North Sea. Institute of Marine Research, Norway | 18 |
| BioChem: Zooplankton collected from the Gully, summer 2006 and 2007 | Kennedy, M.K., Spry, J.A. 2011. BioChem: Zooplankton collected from the Gully, summer 2006 and 2007. OBIS Canada Digital Collections. OBIS Canada, Bedford Institute of Oceanography, Dartmouth, Nova Scotia, Canada, Version 1, Digital, retrieved from http://iobis.org/. | 17 |
| Dalhousie University: Drift net plankton samples collected in Lunenburg Bay, 2005-2006 | Laurent, Arnaud. (2013). Drift net plankton samples collected in Lunenburg Bay, 2005-2006. Version 1 In OBIS Canada Digital Collections. Bedford Institute of Oceanography, Dartmouth, NS, Canada. Published by OBIS, Digital http://www.iobis.org/. | 15 |
| BioChem: Canadian Arctic ThruFlow Study plankton collection | Jim Hamilton. (2014). BioChem: Canadian Arctic ThruFlow Study plankton collection. Version 1 In OBIS Canada Digital Collections. Bedford Institute of Oceanography, Dartmouth, NS, Canada. Published by OBIS, Digital http://www.iobis.org/. | 14 |
| Zooplankton Abundancies White Sea, 1972 | N/A | 14 |
| BioChem: Bedford Basin Monitoring Program zooplankton collection (1991+) | Li, B., Dickie, P. 2012. BioChem: Bedford Basin Monitoring Program zooplankton collection (1991+). OBIS Canada Digital Collections. OBIS Canada, Bedford Institute of Oceanography, Dartmouth, Nova Scotia, Canada, Version 1, Digital, retrieved from http://iobis.org/. | 13 |
| NMFS-COPEPOD: The Global Plankton Database, WEBSEC Sub-Collection | NMFS-COPEPOD: the global plankton database. ONLINE. 2009. Available: http://www.st.nmfs.noaa.gov/plankton/index.html. | 12 |
| Ocean Genome Legacy Collection | Accession ID OGL-#####. The Ocean Genome Legacy Center. Northeastern University. Published on the web at ogl.northeastern.edu/catalog. | 10 |
| Barents Sea Zooplankton – March and May 1998 and July 1999. Zooplankton sampling on board the Norwegian R/V Jan Mayen, program ALV | N/A | 10 |
| Estudio de la comunidad zooplanctónica y fitoplanctónica en los ecosistemas de arrecifes coralinos mesofóticos del Parque Nacional Corales de Profundidad | Criales Hernández M I, Benavides Serrato M, Jerez Guerrero M (2021): Estudio de la comunidad zooplanctónica y fitoplanctónica en los ecosistemas de arrecifes coralinos mesofóticos del Parque Nacional Corales de Profundidad. v1.1. Universidad Industrial de Santander. Dataset/Samplingevent. https://doi.org/10.15472/p70rj0 | 9 |
| Macrobenthos and Meiobenthos Tuktoyaktuk Harbor and Mason Bay 1985-1988 NOGAP | G.E. Hopky, M.J. Lawrence and D.B. Chiperzak (1994) NOGAP B2, Data on the Meio- and Macrobenthos, and related bottom sediments from Tuktoyaktuk Harbour and Mason Bay, N.W.T., March, 1985 to 1988 Canadian Data Report of Fisheries and Ocean Science 939 | 8 |
| IMR Macroplankton surveys | Bakkeplass, K. (2014). IMR Macroplankton surveys. Institute of Marine Research, Norway | 7 |
| Stomach contents of salmonids caught in the Northeastern Pacific Ocean - 1959 and 1960 | LeBrasseur, R.J., Doidge, D.A., 1966. Stomach contents of salmonids caught in the Northeastern Pacific Ocean - 1959 & 1960. Circular, Statistical series, Fisheries Research Board of Canada, Biological Station, Nanaimo, Canada, 21, Vol. 3, 67 pp. | 6 |
| National Museum of Natural History Invertebrate Zoology Collections | National Museum of Natural History, Smithsonian Institution NMNH Invertebrate Zoology Collection Database. National Museum of Natural History, Smithsonian Institution, 10th and Constitution Ave. N.W., Washington, DC 20560-0193, 2001, Version 3.2.04 (0802221). | 6 |
| RMT Trawl catch from the 1995/96 V4 BROKE voyage | Australian Antarctic Data Centre (2017). RMT Trawl catch from the 1995/96 V4 BROKE voyage. | 5 |
| DFO Quebec Region MLI museum collection | Miller R, Nozères C (2025). DFO Quebec Region MLI museum collection. Version 3.19. Fisheries and Oceans Canada. Occurrence dataset. http://iobis.org/mapper/?resource_id=2673 | 4 |
| Zooplankton distribution in the Arctic Ocean based on samples collected at Ice Island T-3 in 1964 | Harding, Gareth C.H.(2016). Zooplankton distribution in the Arctic Ocean based on samples collected at Ice Island T-3 in 1964. Version 2 In OBIS Canada Digital Collections. Bedford Institute of Oceanography, Dartmouth, NS, Canada. Published by OBIS, Digital http://www.iobis.org/. Accessed on –INSERT DATE | 4 |
| RMT Trawl catch from the 1983/84 V5 ADBEX II voyage | Australian Antarctic Data Centre. RMT Trawl catch from the 1983/84 V5 ADBEX II voyage. | 4 |
| UNESCO eDNA expedition in French Austral Lands and Seas (France): December 2022 and April 2023 | Saucède T, Motreuil S, Marschal C, Mignucci A, Marinesque S, Salvatico P, Guéné M, Suominen S, Provoost P, Gillard E, Douvere F, Appeltans W (2024). UNESCO eDNA expedition in French Austral Lands and Seas (France): December 2022 and April 2023. Version 2.0. OBIS Secretariat. Occurrence dataset. https://doi.org/10.25607/qoxzeg | 3 |
| Atlantic Reference Centre Museum of Canadian Atlantic Organisms - Invertebrates and Fishes Data | Van Guelpen, L., 2016. Atlantic Reference Centre Museum of Canadian Atlantic Organisms - Invertebrates and Fishes Data. Version 4 In OBIS Canada Digital Collections. Bedford Institute of Oceanography, Dartmouth, NS, Canada. Published by OBIS, Digital http://www.iobis.org/. | 3 |
| Museums Victoria Marine Invertebrates Collection | Jo Taylor J (2023): Museums Victoria Marine Invertebrates Collection. v1.12. CSIRO National Collections and Marine Infrastructure (NCMI) Information and Data Centre (IDC). Dataset/Occurrence. https://www.marine.csiro.au/ipt/resource?r=nmv_marine_inverts&v=1.12 | 3 |
| Fatty acid profiles of marine consumer from the Southern Hemisphere (1990-2018) | Pethybridge, Heidi; Nichols, Peter; Zhang, Bowen; Virtue, Patti; Meyers, Lauren; Dhurmeea, Zahirah; Marcus, Lara; Ericson, Jessica; Hellessey, Nichole; Every, Sharon; Wheatley, Kathryn; Parrish, Christophere; Eisenmann, Pascale; Baylis, Aliastair; Bradshaw, Corey; Bierwagen, Stacy; Young, Jock; Couturier, Lydie; Rohner, Christophere; Groß, Jasmin; Waugh, Courtney; Phleger, Charles; Jackson, Christine; Jackson, George; Huveneers, Charlie; Bengtson Nash, Susan; Brock, Mina; Mansour, Peter (2022): Fatty acid profiles of marine consumer from the Southern Hemisphere. v1. CSIRO. Data Collection. https://doi.org/10.25919/pdxr-cf66 | 3 |
| Ice core meiofauna during the SIPEX and SIPEX II voyages | Swadling, K. (2017, updated 2018) Ice core meiofauna during the SIPEX and SIPEX II voyages | 2 |
| RMT Trawl catch from the 1986/87 V7 AAMBER voyage | Australian Antarctic Data Centre. RMT Trawl catch from the 1986/87 V7 AAMBER voyage. | 2 |
| SOMBASE – Southern Ocean mollusc database: a tool for biogeographic analysis in diversity and evolution | N/A | 2 |
| DFO Arctic Region Biodiversity of the Benthic Epifauna Trawl Survey from CBS-MEA program (2021-2024) | de Carufel V, Roy V (2025). DFO Arctic Region Biodiversity of the Benthic Epifauna Trawl Survey from CBS-MEA program (2021-2024). Version 1.2. Fisheries and Oceans Canada. Samplingevent dataset. https://ipt.iobis.org/obiscanada/resource?r=cbs_mea&v=1.2 | 2 |
| Trawl Catch and Species Abundance from the 2020 Gulf of Alaska International Year of the Salmon Expedition | Neville, C., Somov, A., Esenkulova, S., & LaForge, R. (2022). Trawl Catch and Species Abundance data from the 2020 Gulf of Alaska International Year of the Salmon Expedition. North Pacific Anadromous Fish Commission. https://doi.org/10.21966/4J6T-GB64 | 2 |
| Tasmanian Museum and Art Gallery Invertebrate Collection - marine records | Byrne C (2023): Tasmanian Museum and Art Gallery Invertebrate Collection - marine records. v1.8. CSIRO National Collections and Marine Infrastructure (NCMI) Information and Data Centre (IDC). Dataset/Occurrence. https://www.marine.csiro.au/ipt/resource?r=ala_co198&v=1.8 | 1 |
| MAREANO - Base-line mapping of epifauna obtained with Beamtrawl | Hassel, A. (2014). MAREANO - Base-line mapping of epifauna obtained with Beamtrawl. Institute of Marine Research, Norway https://doi.org/10.15468/iomgfj | 1 |
| Discovery Collections Midwater Database | Pugh, P. Discovery Collections Midwater Database. National Oceanography Centre, Southampton SO14 3ZH, U.K, 2000. | 1 |
| UFPE Oceanography Zooplankton Research | OBIS (2018): UFPE Oceanography Zooplankton Research. v1. Tropical and Subtropical Western South Atlantic OBIS. Dataset/Occurrence. http://ipt.iobis.org/wsaobis/resource?r=ufpe_oceanography_zooplankton_research&v=1.0 | 1 |
| DFO Arctic Region Biodiversity of the Benthic Infauna Box Core Survey from CBS-MEA program (2021-2023) | de Carufel V, Roy V (2025). DFO Arctic Region Biodiversity of the Benthic Infauna Box Core Survey from CBS-MEA program (2021-2023). Version 1.5. Fisheries and Oceans Canada. Samplingevent dataset. https://ipt.iobis.org/obiscanada/resource?r=cbs_mea_infauna&v=1.5 | 1 |
| Galiano Island BC Canada Marine Zoology 1893–2021 | Simon A, Basman A (2022): Galiano Island BC Canada Marine Zoology 1893–2021. v1.5. Biodiversity Data Journal. Dataset/Occurrence. https://ipt.pensoft.net/resource?r=galiano-data-paper-part-i&v=1.5 | 1 |
| Malacology Collection at the Academy of Natural Sciences of Philadelphia | N/A | 1 |
| Tasmanian Museum and Art Gallery provider for OZCAM - marine records | Webmaster O (2022): Tasmanian Museum and Art Gallery provider for OZCAM - marine records. v1.9. CSIRO National Collections and Marine Infrastructure (NCMI) Information and Data Centre (IDC). Dataset/Occurrence. https://www.marine.csiro.au/ipt/resource?r=tmag_marine&v=1.9 | 1 |
| IMOS National Reference Station (NRS) - Zooplankton Abundance | This material is released under the Creative Commons Attribution 4.0 International licence. The citation in a list of references is: "IMOS [year-of-data-download], [Title], [data-access-URL], accessed [date-of-access]." Any users of IMOS data are required to clearly acknowledge the source of the material in the format described in the acknowledgments section. | 1 |
| UF Invertebrate Zoology | N/A | 1 |

**Table S8:** Biological indicators and their thresholds. For each species a threshold value for aragonite saturation state is provided. For the relevant depth range of each organism the threshold value is used to calculate the threshold diagnostics across the global ocean. percentage (%) area, pre-industrial conditions (PI), 10% decline from pre-industrial (PI-10%), 20% decline from pre-industrial (PI-20%), and year 2020, difference (Δ) in % area between pre-industrial and present day (i.e. expansion of area that has crossed the threshold). Depth ranges used: * surface to 25 m; ^#^ Surface to 200m.

| Species | Life stage | Biological response *(reference)* | Ω_Arag_  threshold | % area passed threshold, PI (global) | % area passed threshold, PI-10% (global) | % area passed  threshold, PI-20%  (global) | % area passed  threshold, 2020  (global) | Δ area  passed threshold (1750 - 2020) (global) |
| --- | --- | --- | --- | --- | --- | --- | --- | --- |
| Pacific oyster (*Magallana gigas*) [*] | larvae | Zero relative larval production (Barton et al., 2012) | 1.75 | 3.2% | 8.8% | 13.9% | 16.6% | 13.4% |
| Olympia oyster (*Ostrea lurida*) [*] | larvae | Sublethal effects on shell growth and shell area at settlement (Hettinger et al., 2012) | 1.4 | 0.1% | 0.3% | 3.2% | 6.3% | 6.2% |
| Eastern oyster (*Crassostrea virginica*) [*] | larvae | Sublethal chronic impact on growth, calcification, malformation (Gobler and Talmage, 2014) | 1.83 | 5.4% | 10.5% | 15.7% | 18.2% | 12.8% |
| California mussel (*Mytilus californianus*) [*] | larvae | Sublethal response in growth, weight and calcification  (Gaylord et al., 2011) | 1.8 | 4.7% | 10.0% | 15.2% | 17.7% | 13.0% |
| Pteropod (*Limacina helicina*) [#] | adult | Mild shell dissolution, high certainty (Bednaršek et al., 2019) | 1.5 | 2.2% | 7.5% | 12.6% | 13.9% | 11.7% |
| Pteropod (*Limacina helicina*) [#] | adult | Severe shell dissolution, high certainty (Bednaršek et al., 2019) | 1.2 | 0.0% | 0.1% | 2.2% | 2.1% | 2.1% |
| Warm-water corals [*] |  | Marginal conditions for growth (Guinotte et al., 2003) | 3.5 | 39.4% | 51.5% | 77.6% | 69.0% | 29.6% |

**Table S9**: Biological indicators and their thresholds. For each species a threshold value for aragonite saturation state is provided. For the relevant depth range of each organism the threshold value is used to calculate the threshold diagnostics across the regional range relevant to each species. Percentage (%) area, pre-industrial conditions (PI), 10% decline from pre-industrial (PI-10%), 20% decline from pre-industrial (PI-20%), and year 2020, difference (Δ) in % area between pre-industrial and present day (i.e. expansion of area that has crossed the threshold), and mean ± SD aragonite saturation state reduction from the pre-industrial conditions that can be made before crossing the threshold. Depth ranges used: ^*^ surface to 25 m; ^#^ Surface to 200m. Region used: ^A^ Global coastal; ^B^ California Current Ecosystem; ^C^ Polar oceans; ^D^ Low latitude regions (40°S to 40°N).

| Species | Life stage | Biological response *(reference)* | Ω_Arag_  threshold | % area passed threshold, PI (regional) | % area passed threshold, PI-10% (regional) | % area passed  threshold,  PI-20%  (regional) | % area passed  threshold,  2020  (regional) | Δ area  passed threshold (1750 - 2020) (regional) | %  reduction from PI  before reaching  threshold |
| --- | --- | --- | --- | --- | --- | --- | --- | --- | --- |
| Pacific oyster (*Magallana gigas*) [*,A] | larvae | Zero relative larval production (Barton et al., 2012) | 1.75 | 4.7% | 9.8% | 14.6% | 17.3% | 12.6% | 52±13% |
| Olympia oyster (*Ostrea lurida*) [*,B] | larvae | Sublethal effects on shell growth and shell area at settlement (Hettinger et al., 2012) | 1.4 | 0.0% | 0.0% | 0.0% | 0.8% | 0.8% | 53±6% |
| Eastern oyster (*Crassostrea virginica*) [*,A] | larvae | Sublethal chronic on growth, calcification, malformation (Gobler and Talmage, 2014) | 1.83 | 7.1% | 11.4% | 16.4% | 19.0% | 11.9% | 48±15% |
| California mussel (*Mytilus californianus*) [*,B], | larvae | Sublethal response in  growth, weight and  calcification (Gaylord et al., 2011) | 1.8 | 6.4% | 10.9% | 15.9% | 18.6% | 12.2% | 51±14% |
| Pteropod (*Limacina helicina*) [#,C] | adult | Mild shell dissolution high confidence score, high certainty (Bednaršek et al., 2019) | 1.5 | 18.7% | 57.1% | 75.9% | 79.5% | 60.8% | 13±16% |
| Pteropod (*Limacina helicina*) [#,C] | adult | Severe shell dissolution high confidence score, high certainty (Bednaršek et al., 2019) | 1.2 | 0.0% | 2.8% | 18.7% | 15.5% | 15.5% | 31±12% |
| Pteropod (*Limacina helicina*) [#,B] | adult | Mild shell dissolution high confidence score, high certainty (Bednaršek et al., 2019) | 1.5 | 0.0% | 8.6% | 36.5% | 42.0% | 42.0% | 24±11% |
| Pteropod (*Limacina helicina*) [#,B] | adult | Severe shell dissolution with high confidence score, high certainty (Bednaršek et al., 2019) | 1.2 | 0.0% | 0.0% | 0.0% | 4.0% | 4.0% | 39±9% |
| Warm-water corals [*,D] |  | Marginal conditions for growth (Guinotte et al., 2003) | 3.5 | 11.2% | 27.5% | 66.8% | 54.0% | 42.8% | 15±9% |

**Table S10**: Biological indicators and their thresholds. As with Table S6, except evaluated at depth 0 m only. B: Polar Oceans, C: California Current Ecosystem. PI: Pre-industrial.

| Species | Aragonite threshold value (ref.) | % area passed threshold, PI | % area passed threshold, PI-10% | % area passed threshold, PI-20% | % area passed threshold, year 2020 | Δ% area passed threshold (1750 - 2020) |
| --- | --- | --- | --- | --- | --- | --- |
| Pteropod^B^ | 1.5 (Bednarsek et al. 2019) | 6.7% | 18.9% | 65.0% | 74.3% | 67.6% |
| Pteropod^B^ | 1.2 (Bednarsek et al. 2019) | 1.5% | 2.7% | 6.7% | 16.4% | 14.9% |
| Pteropod^C^ | 1.5 (Bednarsek et al. 2019) | 0.0% | 0.0% | 0.7% | 0.7% | 0.7% |
| Pteropod^C^ | 1.2 (Bednarsek et al. 2019) | 0.0% | 0.0% | 0.0% | 0.0% | 0.0% |

**Table S11**: Biological indicators and their thresholds. As with Table S7, except evaluated at depth 50 m only. B: Polar Oceans, C: California Current Ecosystem. PI: Pre-industrial.

| Species | Aragonite threshold value (ref.) | % area passed threshold, PI | % area passed threshold, PI-10% | % area passed threshold, PI-20% | % area passed threshold, year 2020 | Δ% area passed threshold (1750 - 2020) |
| --- | --- | --- | --- | --- | --- | --- |
| Pteropod^B^ | 1.5 (Bednarsek et al. 2019) | 8.7% | 26.2% | 65.1% | 75.3% | 66.6% |
| Pteropod^B^ | 1.2 (Bednarsek et al. 2019) | 1.1% | 2.9% | 8.7% | 13.6% | 12.5% |
| Pteropod^C^ | 1.5 (Bednarsek et al. 2019) | 0.0% | 1.6% | 7.7% | 12.9% | 12.9% |
| Pteropod^C^ | 1.2 (Bednarsek et al. 2019) | 0.0% | 0.0% | 0.0% | 0.8% | 0.8% |

**Table S12**: Biological indicators and their thresholds. As with Table S6, except evaluated at depth 100 m only. B: Polar Oceans, C: California Current Ecosystem. PI: Pre-industrial.

| Species | Aragonite threshold value (ref.) | % area passed threshold, PI | % area passed threshold, PI-10% | % area passed threshold, PI-20% | % area passed threshold, year 2020 | Δ% area passed threshold (1750 - 2020) |
| --- | --- | --- | --- | --- | --- | --- |
| Pteropod^B^ | 1.5 (Bednarsek et al. 2019) | 26.3% | 57.2% | 73.5% | 80.0% | 53.7% |
| Pteropod^B^ | 1.2 (Bednarsek et al. 2019) | 7.1% | 10.8% | 26.3% | 23.4% | 16.3% |
| Pteropod^C^ | 1.5 (Bednarsek et al. 2019) | 24.9% | 45.8% | 66.1% | 71.6% | 46.7% |
| Pteropod^C^ | 1.2 (Bednarsek et al. 2019) | 6.3% | 12.4% | 24.9% | 33.0% | 26.7% |

**Table S13**: Biological indicators and their thresholds. As with Table S6, except evaluated at depth 200 m only. B: Polar Oceans, C: California Current Ecosystem. PI: Pre-industrial.

| Species | Aragonite threshold value (ref.) | % area passed threshold, PI | % area passed threshold, PI-10% | % area passed threshold, PI-20% | % area passed threshold, year 2020 | Δ% area passed threshold (1750 - 2020) |
| --- | --- | --- | --- | --- | --- | --- |
| Pteropod^B^ | 1.5 (Bednarsek et al. 2019) | 52.0% | 63.8% | 72.8% | 75.7% | 23.7% |
| Pteropod^B^ | 1.2 (Bednarsek et al. 2019) | 3.7% | 29.6% | 52.0% | 42.7% | 38.9% |
| Pteropod^C^ | 1.5 (Bednarsek et al. 2019) | 99.1% | 100% | 100% | 100 | 0.9% |
| Pteropod^C^ | 1.2 (Bednarsek et al. 2019) | 76.1% | 93.8% | 99.1% | 99.1% | 23.0% |

**Table S14:** Summary statistics for extracted environmental data from OceanSODA-ETHZv1 location matched to warm-water coral reef locations (from UNEP-WCMC, WorldFish Centre, WRI, TNC). (SST = sea surface temperature ; SSS = sea surface salinity; DIC = dissolved inorganic carbon; TA = total alkalinity; Ω_Arag_ = aragonite saturation state; SD = standard deviation; Q1 = lower quartile; Q3 = upper quartile ; min = minimum; max = maximum).

|  | SST | SSS | DIC | TA | pH | pCO_2_ | Ω_Arag_ |
| --- | --- | --- | --- | --- | --- | --- | --- |
| Mean | 28.3 | 33.1 | 1900.3 | 2229.3 | 8.08 | 357.1 | 3.8 |
| SD | 1.6 | 1.6 | 60.7 | 72.0 | 0.02 | 26.5 | 0.2 |
| Median | 28.6 | 32.6 | 1886.7 | 2214.7 | 8.08 | 354.8 | 3.7 |
| Q1 | 27.8 | 32.0 | 1846.9 | 2164.4 | 8.07 | 338.6 | 3.6 |
| Q3 | 29.2 | 34.6 | 1952.0 | 2293.3 | 8.10 | 373.0 | 3.9 |
| 10^th^ percentile | 26.5 | 31.6 | 1834.2 | 2153.6 | 8.05 | 325.6 | 3.5 |
| 90^th^ percentile | 29.8 | 35.3 | 1987.4 | 2327.0 | 8.11 | 391.7 | 4.0 |
| min | 14.0 | 18.4 | 1582.2 | 1764.5 | 7.95 | 274.9 | 2.4 |
| max | 35.9 | 39.9 | 2213.6 | 2554.3 | 8.17 | 533.0 | 5.1 |

**Table S15:** Summary statistics for extracted environmental data from OceanSODA-ETHZv1 location matched to pteropod (*Limacina helicina*) locations (from OBIS). (SST = sea surface temperature ; SSS = sea surface salinity; DIC = dissolved inorganic carbon; TA = total alkalinity; Ω_Arag_ = aragonite saturation state; SD = standard deviation; Q1 = lower quartile; Q3 = upper quartile ; min = minimum; max = maximum).

|  | SST | SSS | DIC | TA | pH | pCO_2_ | Ω_Arag_ |
| --- | --- | --- | --- | --- | --- | --- | --- |
| Mean | 5.5 | 31.5 | 2034.6 | 2187.0 | 8.11 | 347.9 | 1.7 |
| SD | 5.8 | 2.2 | 72.4 | 82.9 | 0.06 | 53.4 | 0.4 |
| Median | 5.7 | 31.9 | 2034.9 | 2197.9 | 8.10 | 348.8 | 1.7 |
| Q1 | -0.3 | 30.8 | 1996.5 | 2170.4 | 8.07 | 318.5 | 1.4 |
| Q3 | 9.4 | 32.6 | 2071.5 | 2219.6 | 8.14 | 376.4 | 2.0 |
| 10^th^ percentile | -1.7 | 29.3 | 1959.6 | 2086.8 | 8.05 | 280.1 | 1.1 |
| 90^th^ percentile | 13.6 | 33.9 | 2128.1 | 2285.6 | 8.19 | 405.7 | 2.3 |
| min | -1.8 | 7.8 | 1257.2 | 1281.9 | 7.51 | 168.3 | 0.3 |
| max | 30.0 | 38.2 | 2295.3 | 2433.9 | 8.37 | 1486.2 | 4.2 |

**Table S16:** Summary statistics for extracted environmental data from OceanSODA-ETHZv1 location matched to pacific oyster (*Magallana gigas*) locations (from OBIS). (SST = sea surface temperature ; SSS = sea surface salinity; DIC = dissolved inorganic carbon; TA = total alkalinity; Ω_Arag_ = aragonite saturation state; SD = standard deviation; Q1 = lower quartile; Q3 = upper quartile ; min = minimum; max = maximum).

|  | SST | SSS | DIC | TA | pH | pCO_2_ | Ω_Arag_ |
| --- | --- | --- | --- | --- | --- | --- | --- |
| Mean | 14.4 | 33.4 | 2030.0 | 2252.7 | 8.10 | 348.3 | 2.5 |
| SD | 5.1 | 2.2 | 67.7 | 95.9 | 0.04 | 34.0 | 0.5 |
| Median | 13.7 | 34.3 | 2043.2 | 2294.6 | 8.10 | 349.0 | 2.5 |
| Q1 | 10.5 | 31.7 | 1989.4 | 2167.6 | 8.07 | 324.3 | 2.1 |
| Q3 | 17.5 | 35.0 | 2075.0 | 2318.0 | 8.13 | 371.4 | 2.8 |
| 10^th^ percentile | 8.4 | 30.6 | 1942.6 | 2130.9 | 8.06 | 305.7 | 1.8 |
| 90^th^ percentile | 21.3 | 35.2 | 2098.8 | 2329.1 | 8.15 | 392.1 | 3.0 |
| min | -0.5 | 5.8 | 1052.7 | 1099.0 | 7.80 | 176.2 | 0.2 |
| max | 32.1 | 38.6 | 2316.5 | 2609.9 | 8.27 | 523.6 | 4.2 |

**Table S17:** Summary statistics for extracted environmental data from OceanSODA-ETHZv1 location matched to mussel (*Mytlius californicus*) locations (from OBIS). (SST = sea surface temperature ; SSS = sea surface salinity; DIC = dissolved inorganic carbon; TA = total alkalinity; Ω_Arag_ = aragonite saturation state; SD = standard deviation; Q1 = lower quartile; Q3 = upper quartile ; min = minimum; max = maximum).

|  | SST | SSS | DIC | TA | pH | pCO_2_ | Ω_Arag_ |
| --- | --- | --- | --- | --- | --- | --- | --- |
| Mean | 13.0 | 32.6 | 2000.8 | 2213.1 | 8.09 | 351.4 | 2.3 |
| SD | 3.8 | 1.3 | 39.5 | 48.2 | 0.03 | 31.1 | 0.4 |
| Median | 12.8 | 32.8 | 2007.3 | 2231.3 | 8.09 | 353.4 | 2.3 |
| Q1 | 10.3 | 32.1 | 1984.2 | 2184.2 | 8.07 | 333.5 | 2.1 |
| Q3 | 15.2 | 33.5 | 2024.9 | 2241.5 | 8.11 | 373.0 | 2.6 |
| 10^th^ percentile | 7.9 | 31.5 | 1955.9 | 2162.3 | 8.05 | 310.9 | 1.9 |
| 90^th^ percentile | 18.0 | 33.6 | 2039.5 | 2248.7 | 8.13 | 388.4 | 2.8 |
| min | 2.6 | 20.1 | 1621.2 | 1758.1 | 7.99 | 243.5 | 1.4 |
| max | 30.6 | 36.5 | 2109.4 | 2386.2 | 8.22 | 462.5 | 4.3 |

**References**

Barton, A., Hales, B., Waldbusser, G.G., Langdon, C., Feelyd, R.A., 2012. The Pacific oyster, Crassostrea gigas, shows negative correlation to naturally elevated carbon dioxide levels: Implications for near-term ocean acidification effects. Limnology and Oceanography 57, 698–710. https://doi.org/10.4319/lo.2012.57.3.0698

Bednaršek, N., Feely, R.A., Howes, E.L., Hunt, B.P.V., Kessouri, F., León, P., Lischka, S., Maas, A.E., Mclaughlin, K., 2019. Systematic Review and Meta-Analysis Toward Synthesis of Thresholds of Ocean Acidification Impacts on Calcifying Pteropods and Interactions With Warming 6, 1–16. https://doi.org/10.3389/fmars.2019.00227

Chau, T.-T.-T., Gehlen, M., Metzl, N., Chevallier, F., 2024. CMEMS-LSCE: a global, 0.25°, monthly reconstruction of the surface ocean carbonate system. Earth Syst. Sci. Data 16, 121–160. https://doi.org/10.5194/essd-16-121-2024

Gaylord, B., Hill, T.M., Sanford, E., Lenz, E.A., Jacobs, L.A., Sato, K.N., Russell, A.D., Hettinger, A., 2011. Functional impacts of ocean acidification in an ecologically critical foundation species. Journal of Experimental Biology 214, 2586–2594. https://doi.org/10.1242/jeb.055939

Gobler, C.J., Talmage, S.C., 2014. Physiological response and resilience of early life-stage Eastern oysters (Crassostrea virginica) to past, present and future ocean acidification. Conservation Physiology 2. https://doi.org/10.1093/conphys/cou004

Gregor, L., Gruber, N., 2021. OceanSODA-ETHZ: A global gridded data set of the surface ocean carbonate system for seasonal to decadal studies of ocean acidification. Earth System Science Data 13, 777–808. https://doi.org/10.5194/essd-13-777-2021

Guinotte, J.M., Buddemeier, R.W., Kleypas, J.A., 2003. Future coral reef habitat marginality: Temporal and spatial effects of climate change in the Pacific basin. Coral Reefs 22, 551–558. https://doi.org/10.1007/s00338-003-0331-4

Hettinger, A., Sanford, E., Hill, T.M., Russell, A.D., Sato, K.N.S., Hoey, J., Forsch, M., Page, H.N., Gaylord, B., 2012. Persistent carry-over effects of planktonic exposure to ocean acidification in the Olympia oyster. Ecology 93, 2758–2768. https://doi.org/10.1890/12-0567.1

Jiang, L., Dunne, J., Carter, B.R., Tjiputra, J.F., Terhaar, J., Sharp, J.D., Olsen, A., Alin, S., Bakker, D.C.E., Feely, R.A., Gattuso, J., Hogan, P., Ilyina, T., Lange, N., Lauvset, S.K., Lewis, E.R., Lovato, T., Palmieri, J., Santana‐Falcón, Y., Schwinger, J., Séférian, R., Strand, G., Swart, N., Tanhua, T., Tsujino, H., Wanninkhof, R., Watanabe, M., Yamamoto, A., Ziehn, T., 2023. Global Surface Ocean Acidification Indicators From 1750 to 2100. J Adv Model Earth Syst 15, e2022MS003563. https://doi.org/10.1029/2022MS003563
